# Blockchain technology embedded in the power battery for echelon recycling selection under the mechanism of traceability

**DOI:** 10.1038/s41598-024-65748-0

**Published:** 2024-07-02

**Authors:** Qingsong Xing, Longxin Ran, Yimeng Li, Baorong Zhou

**Affiliations:** 1 School of Economics and Management, Chongqing Jiaotong University, Chongqing, 400074 China; 2 School of Management, Guizhou University, Guiyang, 550225 China

**Keywords:** Environmental sciences, Environmental social sciences

## Abstract

This paper examines the use of blockchain technology in power battery echelon recycling. The technology helps to improve battery capacity identification and market transaction trust. The study focuses on power battery manufacturers and recycling participants. Two recycling modes are constructed using the Stackelberg game method, and the optimal decision-making of the participating subjects in the two modes of power battery echelon recycling under the embedding of blockchain technology is compared. The influence of each parameter on the optimal decision-making is analyzed. The research findings indicate that the degree of blockchain technology integration rises as the preference coefficient for traceability information increases. When recycling competition is intense and the sensitivity of recycling prices is low, the optimal recycling model for the number of spent power batteries (SPBs) to be recycled is the model in which echelon utilizers do not participate in recycling if the level of cost optimization coefficient embedded in blockchain technology is high, otherwise, it is the model in which echelon utilizers participate in recycling. The profit of power battery manufacturers and echelon utilizers decreases with the increase of the intensity of power battery recycling competition, the cost optimization coefficient of echelon utilizers and the cost optimization coefficient of manufacturers.

## Introduction

In the context of global carbon emission reduction and changes in automobile manufacturing technology, many countries and regions are promoting the development of new energy vehicles. As a result, the global market for new energy vehicles is rapidly expanding, with sales expected to exceed 10 million and a penetration rate of 14% by 2022^1,2^. Power batteries are a core component of new energy vehicles, with a typical service life of 8 years. This means that a significant number of batteries will be retired in the next 5–6 years^3^. The global market for recycling power batteries in new energy vehicles is estimated to exceed 1 trillion watt hours by 2030^4^. Failure to effectively recycle and reuse these batteries will result in a significant waste of resources and serious environmental pollution^5^. Therefore, it is urgent to find ways to fully and efficiently recycle and reuse spent power batteries.

The current recycling of SPBs depends largely on the degree of decay of the battery capacity, and there are mainly two ways of echelon utilization and recycling^6–8^. Specifically, When the remaining capacity of the battery decays to 60–80%, it can be disassembled and reorganized for use in communication base stations, energy storage, and power conditioning scenarios. If the remaining capacity is between 20 and 60% of the original capacity, the battery can be disassembled into individual batteries that can be used for microgrids in series or parallel. If the remaining capacity falls below 20%, the battery should be scrapped and disassembled to obtain the internal battery parts. The recycling method for refining rare metals should be employed^6,9^.

The research on the recycling of SPBs mainly focuses on the subject’s benefit decision-making under the supply chain perspective^10,11^, recycling technology^12^ and its impact on the environment^13^. However, the above research results on the recycling of SPBs, whether at the level of recycling technology or closed-loop supply chain management, its potential premise assumptions that the battery capacity has been attenuated to less than 20% can not be utilized step by step, without taking into account the capacity of retired power batteries can be utilized step by step and can be renewable recycling^14^. In this case, directly dismantling end-of-life vehicles for recycling is a waste of resources and undermines the carbon emission reduction benefits of new energy vehicles.

In this regard, In this regard, scholars have studied the closed-loop supply chain of spent power batteries from the perspectives of supply chain pricing^15–18^, government subsidies^19^ and reward and punishment mechanisms^20^. Yuan et al.^16^ constructed a supply chain pricing model for power batteries considering echelon recycling, and found that the battery lease price is lower under the consideration of echelon recycling; Gu et al.^19^ found that the government would subsidize the echelon utilization consumers only when the remaining capacity of the recycled batteries was high or the echelon recycling utilization rate was low; Savaskan et al.^21^ proposed the manufacturer, retailer and third party recycling models, and found that the retailer recycling model is optimal; Zhang et al.^6^ constructed four joint recycling models, and found that the joint recycling of manufacturers, retailers and third parties is the optimal recycling model when the price sensitivity degree is high and the competition degree is low; Li^22^ discussed EV manufacturer and third party, EV manufacturer and retailer, and retailer and third party joint recycling channel selection, and concluded that the optimal recycling model depends on the intensity of recycling competition and the form of third party economies of scale. Additionally, studies have examined the influence of consumer awareness of environmental responsibility^23^, sensitivity to corporate social responsibility (CSR) efforts^24^, carbon trading policies^25^ and channel competition^26,27^ on the selection of recycling models among various recycling entities.

The study analyzed the relationship between the benefit size of joint recycling mode and the impact of competition intensity on revenue. It is assumed that the traceability information in the SPB recycling market is completely symmetrical, and that recycling participants and consumers are completely certain about the capacity attenuation of SPBs. However, the degradation of SPB capacity is not fully determined due to the possibility of easy tampering of battery capacity degradation data, unclear measurement and identification of degradation gradient data, and low consumer trust in recycling^28^. It is important to consider consumers’ preferences for SPB traceability information. Blockchain, as a distributed ledger technology, offers advantages such as decentralization, openness, transparency, and cryptographic protection^29–31^, which can provide SPB information while preventing information tampering. To address the aforementioned issues, blockchain technology can be utilized to achieve traceability of power battery capacity decay gradient usability and renewable recyclability. This can be done through the implementation of smart contracts, consensus mechanisms, timestamps, and other relevant technologies. The use of blockchain technology can enhance trust between recycling subjects and consumers. Simultaneously, as the extended producer responsibility system is proposed and policies and regulations on the echelon utilization of SPBs are gradually improving, it is increasingly necessary and urgent to study the decision-making process for echelon recycling of power batteries, taking into account the traceability preference embedded in blockchain technology. This statement suggests that the positive supply chain can increase power battery sales and improve the efficiency of SPB recycling, while also reducing the cost of power battery production for manufacturers^32,33^. However, there are few studies in the current academic community that explore blockchain technology embedded in the echelon recycling of SPBs. The few theoretical results that are rich in reference value and significance. For instance, Zhang et al.^28^ established a power battery echelon utilization scheme under blockchain technology and found that the recycling scheme embedded in blockchain technology has a higher increase in socio-economic benefits than the unembedded scheme. Zhou et al.^34^ constructed four recycling models based on blockchain technology and found that the blockchain-embedded recycling model outperformed the non-embedded model. However, the mechanisms that impact the closed-loop supply chain, such as the number of recycling options, the level of competition in recycling channels, the cost of implementing blockchain technology, and the utilization echelon rate, have not been thoroughly studied. This is especially true for joint recycling models that involve multiple parties and consider consumer preferences for traceability information under blockchain technology.

This paper presents a condensed overview of the two modes of direct recycling and non-direct recycling from power battery consumers by echelon utilizers. The main responsibility of the echelon utilizers is to recover SPBs from retailers, third-party recyclers and echelon consumers and process them, with the objective of maximizing the utilization of SPBs and reducing the environmental pollution. If echelon utilizers are directly involved in the recycling process, it will reduce the transfer cost of multiple flows of SPBs. However, the entry of echelon utilizers will lead to increased competition among recycling participants, thus reducing the efficiency of recycling. By comparing the economic benefits of different recycling modes, enterprises can make informed decisions about the most appropriate recycling strategies. Evaluating the recycling quantity of SPBs under different recycling modes can also help to identify a more environmentally friendly and sustainable recycling model. Therefore, it is crucial to study the recycling decisions of each recycling participant in the supply chain under the two power battery recycling modes.

In light of the aforementioned considerations, this paper examines two types of recycling models for SPBs embedded in blockchain technology, taking power battery manufacturers, retailers, echelon utilizers, and third-party recyclers as research objects. The impacts of the intensity of recycling competition and blockchain technology embedded on the number of recycling, as well as the revenue of supply chain main bodies, are explored. The decision model’s validity is further analyzed through numerical simulation, considering consumer traceability preference. The contributions of this work are as follows: (1) This paper presents a Stackelberg game model for the closed-loop supply chain operation problem of power battery recycling. The model includes power battery manufacturers, retailers, third-party recyclers, and echelon utilizers, all embedded with blockchain technology. The study explores the impacts of blockchain technology on recycling quantities and profits of the participating subjects. The aim is to provide a better solution for the SPBs with different attenuation gradients. This article offers theoretical guidance for the recycling of power batteries with different attenuation gradients. It establishes a traceability mechanism that increases the profit and trust of each participating party. (2) This study investigates the optimal recycling models of power battery manufacturers and echelon utilizers under different competition intensities and echelon utilization rates, considering consumers’ traceability preferences. The impacts of these factors on the number of SPBs recycled, as well as the profits of manufacturers and echelon utilizers, are further analyzed to provide theoretical support for the implementation of the extended producer responsibility system by manufacturers of power batteries and new energy vehicles, as well as for joint recycling cooperation between manufacturers and echelon utilizers of power batteries. This study provides theoretical support for the implementation of the extended producer responsibility system by manufacturers of power batteries and new energy vehicles and for the cooperation with echelon users in the recycling of power batteries.

## The model

### Problem description

**Figure 1:**
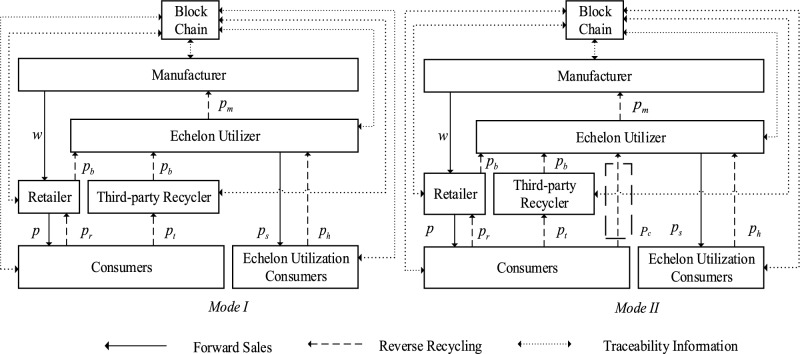
Blockchain technology embedded in power battery echelon recycling mode under traceability mechanism.

After power battery manufacturers embed blockchain technology, it has two main benefits. Firstly, it makes it possible to clearly identify the remaining capacity of SPBs and their recyclability, which reduces the processing cost of power batteries’ Echelon utilization and improves market demand. Secondly, the embedding of blockchain technology in power battery recycling systems provides strong regulatory support for establishing traceability mechanisms and ensuring timely, true, and accurate uploading of traceability information. Embedding blockchain technology in the power battery recycling system to establish a real-time traceability mechanism for trustworthy transactions requires the cooperation of participating subjects and significant investment in resources and technological research by power battery manufacturers, which enables data openness, transparency, and decentralization. Through this traceability mechanism, each participant in the recycling process can record and store information on the production, sales, and recycling of each level and subject of the closed-loop supply chain using the blockchain consensus and trust mechanisms. This information can be used for traceability management of the closed-loop supply chain for the recycling of power batteries for echelon utilization. However, power battery manufacturers must consider market demand resulting from the embedding of blockchain technology under the traceability mechanism, the reduced cost of echelon utilization processing, and the cost effect of technology investment, so as to realize the optimization of decision-making at the level of embedding blockchain technology. Based on this, there are two typical modes of embedding blockchain technology into a closed-loop supply chain consisting of a power battery manufacturer, a retailer, an echelon utilizer, and a third-party recycler under the traceability mechanism. (1) Model $I$ involves joint recycling by retailers and third-party recyclers. For example, Ningde Times relies on 4S stores and Guangdong Bangpu Recycling Technology Co. to recycle retired power batteries^35^. (2) Model $II$ involves joint recycling efforts between retailers, third-party recyclers, and echelon utilizers. For instance, Shanghai Tesla Ltd. relies on 4S stores to recycle SPBs and has also signed a recycling agreement with Shanghai Huadong Wrecking Co. and Shanghai Greenmax Co. The closed-loop supply chain structure is illustrated in Fig. 1.

The paper addresses the following problem:

1. What are the optimal pricing decisions and optimal profits of each subject in the supply chain under the two recycling models?
2. How do key parameters such as the level of blockchain technology embedded, consumers’ preference for traceability information, and the intensity of competition among the recycling participants of SPBs affect the decision-making results?
3. What are the results of the comparative analysis of the optimal recycling quantity of SPBs for each recycling participant in the supply chain under the two recycling models?
4. What is the sensitivity of the optimal decision-making results under different conditions for each subject in the supply chain under the two recycling models?

In the forward supply chain, manufacturers produce new energy power batteries using newly purchased raw materials or recycled materials extracted from power batteries that cannot be used in the echelon, while blockchain technology is embedded in the power batteries, which are then sold to consumers through retailers. Blockchain technology can record all sales and recycling information of power batteries in a closed-loop supply chain. The information can be traced in the block according to the timestamp to ensure transparency and information sharing. Smart contract technology can enhance transaction speed for participating entities in power battery recycling, saving time and cost. In the reverse supply chain, retailers and third-party recyclers recycle SPBs. These recycling participants then resell the recycled SPBs to echelon utilizers. The echelon utilizer identifies SPBs based on the information in the block and divides them into two categories: batteries that can be used for echelon purposes and those that cannot, based on their remaining capacity. It processes the batteries that can be used for echelon purposes, sells them as products for echelon use in the echelon use market, and is responsible for collecting the batteries that cannot be used for echelon purposes from the echelon use market. Finally, the echelon user will sell the non-echelon usable power battery back to the manufacturer for raw material extraction. It is important to note that in the entire closed-loop supply chain of new energy vehicles, retailers (4S stores) and third-party recyclers are closer to consumers and have advantages in recycling. Therefore, such recycling channels as retailer recycling and third-party recycler recycling are essential^6^.

### Basic assumptions

#### Assumption 1

Manufacturers sell and recycle the same type of power battery. The leaders and echelon utilizers are the manufacturers, while third-party recyclers and retailers are the followers. Batteries not used by echelon refer to the portion of spent power batteries that have not undergone echelon utilization, as well as the recycled materials such as lithium, nickel, and slam extracted from these batteries. The quality of power batteries produced using recycled materials is equivalent to those produced using raw materials^6^.

#### Assumption 2

Blockchain technology relies on smart contracts, consensus mechanisms, timestamps, and other technologies to achieve data transparency and sharing in the recycling process of spent power batteries. This enhances consumer trust while satisfying their traceability preferences, resulting in new market demand^3^. The demand for power batteries is related to consumers’ preference for traceability information under blockchain embedding. The demand function for power batteries is represented by $D = a - bp + kt$ ^23^, where $a$ denotes the potential market demand when the retail price is zero and blockchain technology is not embedded, $b$ denotes the coefficient of sensitivity of consumers to the retail price of power batteries, $p$ denotes the retail price, $k$ denotes the preference of consumers for traceability information under blockchain embedding, and $t$ denotes the level of blockchain technology embedding.

#### Assumption 3

The EU’s New Battery Regulation, issued in July 2023, requires battery manufacturers to extend their producer responsibility to include organizing the classification, recovery, regeneration, and recycling of spent power batteries, as well as echelon use activities. The manufacturer fully bears the input cost of blockchain technology embedded in power batteries, which is a quadratic function of the level of blockchain technology embedded. The input cost of blockchain technology embedded in power battery is fully borne by the manufacturer and is a quadratic function of the level of blockchain technology embedded, i.e., $$C{ = }\frac{1}{2}\mu t^{2}$$ ^17^, $$\mu$$ denotes the investment cost coefficient of blockchain technology embedded, and $$t$$ denotes the level of blockchain technology embedded. Once the power battery manufacturer is integrated into the blockchain, the cost of production for both the manufacturer and the echelon utilizer will decrease. This is due to the increase in consumer trust and the reduction of market transaction costs. The cost optimization coefficients will be represented by $$\varphi$$ and $$\rho$$$$\left( {0 < \varphi < 1,0 < \rho < 1} \right)$$, respectively. Smaller values of $$\varphi$$ and $$\rho$$ indicate greater cost optimization ability, while larger values indicate the opposite^28^.

#### Assumption 4

The recycling quantity of spent power batteries is $Q_{j}^{i} = q + mp_{j} - np_{l}$, $m > n > 0$, $i \in \left\{ {I,II} \right\}$, $j,l \in \left\{ {r,c,t} \right\}$,$j \ne l$, where mode $I$ and mode $II$ denote two different modes, respectively. Retailers, echelon utilizers, and third-party recyclers are denoted by $r$,$c$,$t$, respectively. $q$ denotes the recycling quantity when the recycling price and the price of the competitive channel are zero. $m$ denotes the sensitivity coefficient of the recycling price, $p_{j}$ denotes the recycling price, while $n$ denotes the price sensitivity coefficient of the competitive channel, and $p_{l}$ denotes the competitive channel’s recycling price. The total quantity of spent power batteries that have been recycled is represented by $Q = Q_{r} + Q_{c} + Q_{t}$ ^32,36^. Among the spent power batteries that have been recycled, a portion of them (denoted as $\beta$) can be utilized for echelon use. These batteries are processed into echelon use products, with the remaining capacity ranging between 20 and 80%, and are sold to consumers for echelon use. The batteries that can not be utilized for echelon use are all resold to the manufacturers to be directly dismantled and recycled (the remaining capacity of the batteries that can not be utilized for echelon use is less than 20% and also contains batteries that are recycled from consumers for echelon use). Non-recyclable batteries are sold to manufacturers for direct dismantling and recycling if they have less than 20% residual capacity. This includes batteries recycled from consumers who use them in a gradient. The recycled materials extracted from these batteries can be used to produce new batteries at a lower cost than using new materials, resulting in $c_{n} - c_{r} \rho > 0$. Meanwhile, during the process of recycling materials into new power batteries, there is inevitably some loss. As a result, there is a residual rate of $\alpha (0 < \alpha < 1)$.

### Parameters of the model

Table 1 displays the relevant parameters of the model:

**Table 1:** Related parameters and their description.

Parameter	Meaning
$\mu$	Cost factor for investing in blockchain technology
$k$	Consumer preference coefficient for traceable information with blockchain embedding
$\varphi$	Processing cost optimization factor for the echelon utilization power battery after blockchain embedding
$\rho$	Cost optimization factor for power battery production using recycled materials after blockchain embedding
$a$	Potential market demand
$c_{p}$	Processing cost of power batteries for recycling
$p_{s}$	Selling price of echelon utilization power batteries
$p_{h}$	Recycling price of echelon utilization power batteries recovered by echelon utilizers from echelon utilization consumers
$b$	Sensitivity factor of power battery sales price to sales quantity
$q$	The amount of power batteries recycled when the recycling price for such batteries is zero
$m$	Sensitivity factor of power battery recycling quantity to recycling price
$n$	Recycling price sensitivity coefficients of power battery recycling quantities to recycling competition channels
$c_{n}$	The unit cost of batteries produced by power battery manufacturers using new materials
$c_{r}$	The unit cost of batteries produced by power battery manufacturers using recycled materials
$\beta$	SPBs can be used echelonally
$\alpha$	The residual rate of power batteries with less than 20% electric capacity entering the dismantling and utilization stage
$w$	Unit wholesale price of power batteries
$p$	Unit retail price of power batteries
$t$	Embedding level of blockchain technology
$p_{r}$	Recycling price for retailers recycling SPBs
$p_{m}$	Transfer price for manufacturers collecting SPBs from echelon utilizers
$p_{b}$	Transfer price for collection of SPBs from retailers or third-party recyclers by echelon utilizers
$p_{t}$	Recycling prices for SPBs by third-party recyclers
$p_{c}$	Recycling price for the recycling of SPBs from consumers by echelon utilizers
$D$	Demand function of power battery
$C$	Cost function for investing in blockchain technology
$\pi_{j}^{i}$	The profit function of $j$ in model $i$, where $i = I,II$ represents joint recycling by retailers and third-party recyclers, and joint recycling by retailers, third-party recyclers, and echelon utilizers, respectively
$Q_{j}^{i}$	The recycling quantity function for $j$ in Model $i$, where $i = I,II$, $j = r,c,t$, has the same meaning as previously defined

## Model establishment and solution

### Model $I$: joint recycling by the retailer and the third-party recycler

This model involves the power battery manufacturer determining parameters $w$, $p_{m}$, and $t$, and the echelon utilizer responding by determining the transfer price of the power battery, $p_{b}$. The retailer (4S store) and the third-party recycler respond to the decisions made by the manufacturer and the echelon utilizer by determining parameters $p$, $p_{r}$, and $p_{t}$. Based on the aforementioned parameter design and basic assumptions, the respective profits of the third-party recycler, the retailer, the manufacturer, and the echelon utilizer can be derived using the following functions:

1 $$\pi_{t}^{I} { = }\left( {p_{b} - p_{t} } \right)Q_{t}^{I}$$

2 $$\pi_{r}^{I} = \left( {p - w} \right)D + \left( {p_{b} - p_{r} } \right)Q_{r}^{I}$$

3 $$\pi_{m}^{I} { = }\left( {w - c_{n} } \right)D + \left( {c_{n} - \rho {\text{c}}_{r} - p_{m} } \right)\left( {Q_{r}^{I} + Q_{t}^{I} } \right)\left( {1 - \beta + \beta \alpha } \right) - \mu t^{2} /2$$

4 $$\begin{aligned} \pi_{c}^{I} { = } & \beta {\text{Q}}_{r}^{I} \left( {p_{s} - \varphi {\text{c}}_{p} - p_{b} - p_{h} + \alpha {\text{p}}_{m} } \right) + \left( {1 - \beta } \right)Q_{r}^{I} \left( {p_{m} - p_{b} } \right) \\ & + \beta Q_{t}^{I} \left( {p_{s} - \varphi c_{p} - p_{b} - p_{h} + \alpha p_{m} } \right) + \left( {1 - \beta } \right)Q_{t}^{I} \left( {p_{m} - p_{b} } \right) \\ \end{aligned}$$

In formulas (1)–(4), the manufacturer profit function is defined as the sum of the net wholesale battery profit and the cost savings from producing batteries from renewable materials, minus the blockchain technology embedding cost. The retailer’s profit function is the net profit from the sale of batteries, plus the net proceeds from recycling of SPBs. The third-party recycler’s profit function is the net proceeds from recycling of SPBs. The profit function of the echelon utilizers is comprised of two components: the net profit derived from processing SPBs recovered from retailers and third-party recyclers into recycled power batteries and selling them, and the net profit derived from recycling non-echelon recyclable power batteries to manufacturers.

#### Proposition 1

In model $I$, when $\mu > {{k^{2} } \mathord{\left/ {\vphantom {{k^{2} } {4b}}} \right. \kern-0pt} {4b}}$, the optimal decisions of the manufacturer, retailer, echelon utilizer, and third-party recycler are as follows:

5 $$t^{{I^{*} }} { = }\left( { - c_{n} b + a} \right)k{/}\left( {4\mu b - k^{2} } \right)$$

6 $$p_{b}^{{I^{*} }} = \left( {\left( {m - n} \right)F - 3q} \right)/\left( {4m - 4n} \right)$$

7 $$w^{{I^{*} }} { = }\left( {\left( {2c_{n} b + 2a} \right)\mu - c_{n} k^{2} } \right)/\left( {4\mu b - k^{2} } \right)$$

8 $$p^{{I^{*} }} { = }\left( {\left( {c_{n} b + 3a} \right)\mu - c_{n} k^{2} } \right){/}\left( {4\mu b - k^{2} } \right)$$

9 $$p_{r}^{{I^{*} }} = \left( {F{\mkern 1mu} m^{2} - Fnm - 7qm + 4nq} \right)/\left( {8m^{2} - 12mn + 4n^{2} } \right)$$

10 $$p_{t}^{{I^{*} }} = \left( {F{\mkern 1mu} m^{2} - Fnm - 7qm + 4nq} \right)/\left( {8m^{2} - 12mn + 4n^{2} } \right)$$

11 $$\begin{aligned} p_{m}^{{I^{*} }} & = \left( { - \left( {m - n} \right)\left( {\left( { - \alpha + 1} \right)c_{n} + \rho \left( {\alpha - 1} \right)c_{r} - c_{p} \varphi + p_{s} - p_{h} } \right)\beta + \left( { - c_{r} \rho + c_{n} } \right)m } \right. \\ & \quad \left. {+ \left( {c_{r} \rho - c_{n} } \right)n - q} \right)/2\left( {m - n} \right)\left( {1 + \left( {\alpha - 1} \right)\beta } \right) \end{aligned}$$

where $F = \left( {c_{n} - \rho c_{r} } \right)(1 + \beta \left( {\alpha - 1} \right)) + p_{s} - c_{p} \varphi - p_{h} > 0$, it can be seen by the expression of $F$ that if the residual rate $\alpha$ of the power battery for echelon utilization into the dismantling and utilization stage and the rate $\beta$ of the recycled SPBs that can be echelon utilized in the recycled SPBs are larger, $F$ is larger. If the optimization coefficient $\rho$ of the processing cost of blockchain technology embedded in the echelon utilization of the power battery and the optimization coefficient $\varphi$ of the cost of the production of the power battery by using recycled material are smaller, $F$ will be larger.

#### Proof

See Online Appendix A.

### Model $II$: joint recycling by the retailer, the echelon utilizer and the third-party recycler

In this model, the power battery manufacturer first determines $w$, $p_{m}$ and $t$, and the echelon utilizer responds to the manufacturer by determining the transfer price of the power battery $p_{b}$ and the recycling price $p_{c}$. The retailer and the third-party recycler respond to the decisions of the manufacturer and the echelon utilizer by determining $p$, $p_{r}$ and $p_{t}$. Based on the aforementioned parameter design and scenario assumptions, the profit functions of the third-party recycler, the retailer, the manufacturer, and the echelon utilizer are obtained as follows:

12 $$\pi_{t}^{II} { = }\left( {p_{b} - p_{t} } \right)Q_{t}^{II}$$

13 $$\pi_{r}^{II} = \left( {p - w} \right)D + \left( {p_{b} - p_{r} } \right)Q_{r}^{II}$$

14 $$\pi_{m}^{II} = \left( {w - c_{n} } \right)D + \left( {c_{n} - \rho c_{r} - p_{m} } \right)\left( {Q_{r}^{II} + Q_{t}^{II} + Q_{c}^{II} } \right)\left( {1 - \beta + \beta \alpha } \right) - \mu t^{2} /2$$

15 $$\begin{aligned} \pi_{c}^{II} { = } & \beta ({\text{Q}}_{r}^{II} + {\text{Q}}_{t}^{II} )\left( {p_{s} - \varphi {\text{c}}_{p} - p_{b} - p_{h} + \alpha {\text{p}}_{m} } \right) + \left( {1 - \beta } \right)Q_{c}^{II} \left( {p_{m} - p_{c} } \right) \\ & + \beta Q_{c}^{II} \left( {p_{s} - \varphi c_{p} - p_{c} - p_{h} + \alpha p_{m} } \right) + \left( {1 - \beta } \right)(Q_{r}^{II} + Q_{t}^{II} )\left( {p_{m} - p_{b} } \right) \\ \end{aligned}$$

In formulas (12)–(15), the manufacturer profit function is defined as the sum of the net profit from wholesale batteries and the cost savings from producing batteries from renewable materials, minus the cost of embedding the blockchain technology. The retailer profit function is defined as the net profit derived from battery sales and the net gain from recycled SPBs. The third-party recycler profit function is the net revenue generated from recycled SPBs. The profit function of the echelon utilizers comprises two elements: the net profit derived from processing the SPBs recovered directly from consumers into echelon utilizable batteries and selling them, and the net profit from recycling the non-echelon utilizable batteries to the manufacturers, as compared to Model I. Please find specific revisions on pages 8 and 9 of the revised manuscript.

#### Proposition 2

In model $II$, when $m > 2n$ and $\mu > {{k^{2} } \mathord{\left/ {\vphantom {{k^{2} } {4b}}} \right. \kern-0pt} {4b}}$ are satisfied, the optimal decisions of the manufacturer, the retailer, the echelon utilizer, and the third-party recycler are:

16 $$t^{{II^{*} }} { = }\left( {\left( { - c_{n} b + a} \right)k} \right){/}\left( {4\mu b - k^{2} } \right)$$

17 $$p_{b}^{{II^{*} }} = \left( {\left( {m - 2n} \right)F - 3q} \right)/\left( {4m - 8n} \right)$$

18 $$w^{{II^{*} }} { = }\left( {\left( {2c_{n} b + 2a} \right)\mu - c_{n} k^{2} } \right){/}\left( {4\mu b - k^{2} } \right)$$

19 $$p^{{II^{*} }} { = }\left( {\left( {c_{n} b + 3a} \right)\mu - c_{n} k^{2} } \right){/}\left( {4\mu b - k^{2} } \right)$$

20 $$p_{c}^{{II^{*} }} = \left( {\left( {m - 2n} \right)F - 3q} \right)/\left( {4m - 8n} \right)$$

21 $$p_{r}^{{II^{*} }} = \left( {F{\mkern 1mu} m^{2} - Fnm - 2F{\mkern 1mu} n^{2} - 7qm + 5nq} \right)/\left( {8m^{2} - 20mn + 8n^{2} } \right)$$

22 $$p_{t}^{{II^{*} }} = \left( {F{\mkern 1mu} m^{2} - Fnm - 2F{\mkern 1mu} n^{2} - 7qm + 5nq} \right)/\left( {8m^{2} - 20mn + 8n^{2} } \right)$$

23 $$\begin{aligned} p_{m}^{{II^{*} }} & = \left( { - \left( {m - 2n} \right)\left( {\left( { - \alpha + 1} \right)c_{n} + \rho \left( {\alpha - 1} \right)c_{r} - c_{p} \varphi + p_{s} - p_{h} } \right)\beta }\right. \\ & \quad \left. { + \left( { - c_{r} \rho + c_{n} } \right)m + \left( {c_{r} \rho - c_{n} } \right)n - q} \right)/2\left( {m - 2n} \right)\left( {1 + \left( {\alpha - 1} \right)\beta } \right) \end{aligned}$$

The proof is similar to model $I$ and will not be repeated here.

## Analysis and comparison of equalization results

Propositions 1 and 2 provide the optimal decision results for manufacturers, retailers, echelon utilizers, and third-party recyclers under model $I$ and mode $II$, respectively. By analyzing the optimal decisions and benefits of the two recycling models, the following inferences can be made.

### Corollary 1

The relationship between the manufacturer’s optimal wholesale price, the level of blockchain technology embedding, the retailer’s optimal retail price, and the consumer’s optimal quantity demanded under the two recycling models is shown below:

$$w^{{I^{*} }} = w^{{II^{*} }} ,\;t^{{I^{*} }} = t^{{II^{*} }} ,\;p^{{I^{*} }} = p^{{II^{*} }} ,\;D^{{I^{*} }} = D^{{II^{*} }}$$

### Proof

See Online Appendix B.

Corollary 1 demonstrates that the optimal wholesale price, level of blockchain technology embedding, optimal retail price, and optimal quantity demanded by consumers for manufacturers are the same under the two different recycling modes, i.e., the forward supply chain sales decision is not related to the choice of the reverse supply chain recycling mode, and power battery sales and recycling are two relatively independent businesses, and power battery manufacturers are not affected by the recycling mode when making sales decisions.

### Corollary 2

In the both recycling modes, the relationship between the variation of manufacturer’s profit with the increase of the residual rate $\alpha$ of power battery entering the disassembly and utilization stage, the investment cost coefficient $\mu$ embedded in the blockchain technology, the cost optimization coefficient $\rho$ of using recycled materials to produce power batteries, and the consumer’s preference $k$ of traceability information exists as follows: ${{\partial \pi_{m}^{I*} } \mathord{\left/ {\vphantom {{\partial \pi_{m}^{I*} } {\partial \alpha }}} \right. \kern-0pt} {\partial \alpha }} > 0$, ${{\partial \pi_{m}^{II*} } \mathord{\left/ {\vphantom {{\partial \pi_{m}^{II*} } {\partial \alpha }}} \right. \kern-0pt} {\partial \alpha }} > 0$; ${{\partial \pi_{m}^{I*} } \mathord{\left/ {\vphantom {{\partial \pi_{m}^{I*} } {\partial \mu }}} \right. \kern-0pt} {\partial \mu }} < 0$, ${{\partial \pi_{m}^{II*} } \mathord{\left/ {\vphantom {{\partial \pi_{m}^{II*} } {\partial \mu }}} \right. \kern-0pt} {\partial \mu }} < 0$; ${{\partial \pi_{m}^{I*} } \mathord{\left/ {\vphantom {{\partial \pi_{m}^{I*} } {\partial \rho }}} \right. \kern-0pt} {\partial \rho }} < 0$, ${{\partial \pi_{m}^{II*} } \mathord{\left/ {\vphantom {{\partial \pi_{m}^{II*} } {\partial \rho }}} \right. \kern-0pt} {\partial \rho }} < 0$; ${{\partial \pi_{m}^{I*} } \mathord{\left/ {\vphantom {{\partial \pi_{m}^{I*} } {\partial k}}} \right. \kern-0pt} {\partial k}} > 0$, ${{\partial \pi_{m}^{II*} } \mathord{\left/ {\vphantom {{\partial \pi_{m}^{II*} } {\partial k}}} \right. \kern-0pt} {\partial k}} > 0$.

### Proof:

See Online Appendix C.

Corollary 2 demonstrates that the profit of the manufacturer increases as the residual rate $\alpha$ of the power battery entering the dismantling and utilization stage increases in both models. the larger $\alpha$ indicates that the manufacturer obtains more recycled materials, and since the cost of using recycled materials to produce new power batteries is lower than that of using raw materials^34^, the more recycled materials that enter the dismantling and utilization stage, the more profit the manufacturer obtains. The profit of manufacturers decreases as the investment cost coefficient $\mu$ embedded in blockchain technology increases in both models. This is because a higher value of $\mu$ results in a higher investment cost embedded in the blockchain technology, leading to lower profits for the manufacturer. The manufacturer’s profit decreases in both modes as the cost optimization coefficient $\rho$ for using recycled materials to produce power batteries increases after blockchain embedding. The manufacturer must evaluate and test recovered non-echelon utilization power batteries to screen out recycled materials for use in producing power batteries. This is necessary because non-echelon utilization power batteries contain waste materials. Therefore, embedding blockchain technology can reduce the cost of using recycled materials to produce power batteries for manufacturers to a certain extent. The cost optimization ability embedded in blockchain technology is expressed by $1 - \rho$. The larger $\rho$ is, the higher the cost of using recycled materials $\rho {\text{c}}_{r}$ to produce power batteries is, and therefore the lower the profit of the manufacturer $c_{n} - \rho {\text{c}}_{r}$ is. Manufacturers’ profits in both modes increase with the enhancement of consumers’ preference for traceability information $k$, which stems from the fact that the embedding of blockchain technology under the traceability mechanism can make the gradient of the remaining capacity degradation of SPBs clearly labeled, reduce the information asymmetry in the closed-loop supply chain of the gradient recycling, satisfy the consumers’ preference for traceability information, enhance the consumers’ sense of trust in the transaction, and expand the market breadth for the demand for the gradient utilization, which in turn enhances the manufacturers’ market share and increases the manufacturers’ profits.

### Corollary 3

The transfer prices of manufacturers and echelon utilizers for the two recycling models are related as follows:

1. $$p_{m}^{{I^{*} }} > p_{m}^{{II^{*} }} ,\;p_{b}^{{I^{*} }} > p_{b}^{{II^{*} }}$$;
2. When $2n < m < m_{1}$, if $F > F_{3}$, then $p_{r}^{{I^{*} }} = p_{t}^{{I^{*} }} < p_{r}^{{II^{*} }} = p_{t}^{{II^{*} }}$; if $F_{1} < F < F_{3}$, then $p_{r}^{{I^{*} }} = p_{t}^{{I^{*} }} > p_{r}^{{II^{*} }} = p_{t}^{{II^{*} }}$;
3. When $m > m_{1}$, if $F > F_{2}$, then $p_{r}^{{I^{*} }} = p_{t}^{{I^{*} }} < p_{r}^{{II^{*} }} = p_{t}^{{II^{*} }}$; if $F_{1} < F < F_{2}$, then $p_{r}^{{I^{*} }} = p_{t}^{{I^{*} }} > p_{r}^{{II^{*} }} = p_{t}^{{II^{*} }}$;

### Proof

See Online Appendix D.

Corollary 3 demonstrates that: (1) the transfer prices of manufacturers and echelon utilizers in Mode $I$ are higher than in Mode $II$ due to the diversity of scenarios used by echelon utilizers and the low saturation of battery capacity in Mode $II$. This results in higher recycling prices than those of retailers and third-party recyclers in Mode $I$. As a result, consumers are more likely to choose the recycling channels of echelon utilizers, and retailers and third-party recyclers will increase the recycling price to gain access to the recycling market. This will lead to an improvement in the market supply of SPBs in Mode $II$, which will be higher than in Mode $I$. In this case, as the market supply increases, the recycling price starts to fall. Consequently, the transfer price will also decrease, leading to an increase in the profit of each recycling participant.

(2) when the recycling channel has a high competitive intensity and the sensitivity of consumers’ recycling is low, if the cost optimization coefficient embedded in the block technology is smaller, i.e., the cost optimization ability is larger, the recycling price of Mode $I$ is lower than the recycling price of Mode $II$ If the cost optimization factor is large, meaning that the cost optimization capacity is small, the recovery price Model $I$ is higher than that of Model $II$. This is due to the enhanced cost optimization capacity of blockchain technology, which results in higher recycling prices for echelon utilizers compared to that of retailers and third-party recyclers in Mode $II$. Although the competition sensitivity factor of the recycling channel is larger, due to the smaller recycling price sensitivity factor, the increase of recycling prices by retailers and third-party recyclers will not have a significant impact on the quantity of recycling. Consequently, compared with Mode $I$, the recycling prices of retailers and third-party recyclers in Mode $II$ are higher. However, the weaker cost optimization ability does not confer an advantage on echelon utilizers in recycling prices in Mode $II$. Furthermore, due to the smaller recycling price sensitivity coefficients, retailers and third-party recyclers choose to lower their recycling prices in order to offset the loss of revenues resulting from the reduction in recycling quantities as a consequence of the entry of echelon utilizers into the recycling market.

(3) When the competition in the recycling channel is low and consumers are more sensitive to recycling, the recycling price of Model $I$ is lower than that of Model $II$ if the cost optimization coefficient is smaller. Conversely, if the cost optimization coefficient is larger, the recycling price of Model $I$ is higher than that of Model $II$. This is because the stronger cost-optimization capability makes the echelon utilizer’s recycling price significantly higher than that of the retailer and third-party recycler in Mode $II$, and although the competitive sensitivity coefficient of the recycling channel is smaller, the amount of recycling changes significantly when the retailer and third-party recycler increase their recycling price due to the larger recycling price sensitivity coefficient, so the retailer and third-party recycler will increase their recycling price in Mode $II$ and thus higher than in Model $I$. When the cost optimization ability is weak, the recycling price of the echelon utilizer in Model $II$ is not significantly different from that of the retailer and the third-party recycler, and although the recycling price sensitivity coefficient is larger, the retailer and the third-party recycler will choose to lower the recycling price to reduce the profit loss.

### Corollary 4

The optimal recycling quantities for third-party recyclers and retailers under the two recycling models have a relationship as follows:

1. $$Q_{t}^{{I^{*} }} = Q_{r}^{{I^{*} }} > Q_{t}^{{II^{*} }} = Q_{r}^{{II^{*} }}$$;
2. When $$1 > \alpha > \alpha_{1}$$, $${{\partial (Q_{r}^{{I^{*} }} - Q_{r}^{{II^{*} }} )} \mathord{\left/ {\vphantom {{\partial (Q_{r}^{{I^{*} }} - Q_{r}^{{II^{*} }} )} {\partial \beta }}} \right. \kern-0pt} {\partial \beta }} = {{\partial (Q_{t}^{{I^{*} }} - Q_{t}^{{II^{*} }} )} \mathord{\left/ {\vphantom {{\partial (Q_{t}^{{I^{*} }} - Q_{t}^{{II^{*} }} )} {\partial \beta }}} \right. \kern-0pt} {\partial \beta }} > 0$$, when $$0 < \alpha < \alpha_{1}$$, $${{\partial (Q_{r}^{{I^{*} }} - Q_{r}^{{II^{*} }} )} \mathord{\left/ {\vphantom {{\partial (Q_{r}^{{I^{*} }} - Q_{r}^{{II^{*} }} )} {\partial \beta }}} \right. \kern-0pt} {\partial \beta }} = {{\partial (Q_{t}^{{I^{*} }} - Q_{t}^{{II^{*} }} )} \mathord{\left/ {\vphantom {{\partial (Q_{t}^{{I^{*} }} - Q_{t}^{{II^{*} }} )} {\partial \beta }}} \right. \kern-0pt} {\partial \beta }} < 0$$;
3. $${{\partial (Q_{r}^{{I^{*} }} - Q_{r}^{{II^{*} }} )} \mathord{\left/ {\vphantom {{\partial (Q_{r}^{{I^{*} }} - Q_{r}^{{II^{*} }} )} {\partial \rho }}} \right. \kern-0pt} {\partial \rho }} = {{\partial (Q_{t}^{{I^{*} }} - Q_{t}^{{II^{*} }} )} \mathord{\left/ {\vphantom {{\partial (Q_{t}^{{I^{*} }} - Q_{t}^{{II^{*} }} )} {\partial \rho }}} \right. \kern-0pt} {\partial \rho }} < 0$$; $${{\partial (Q_{r}^{{I^{*} }} - Q_{r}^{{II^{*} }} )} \mathord{\left/ {\vphantom {{\partial (Q_{r}^{{I^{*} }} - Q_{r}^{{II^{*} }} )} {\partial \varphi }}} \right. \kern-0pt} {\partial \varphi }} = {{\partial (Q_{t}^{{I^{*} }} - Q_{t}^{{II^{*} }} )} \mathord{\left/ {\vphantom {{\partial (Q_{t}^{{I^{*} }} - Q_{t}^{{II^{*} }} )} {\partial \varphi }}} \right. \kern-0pt} {\partial \varphi }} < 0$$

### Proof

See Online Appendix E.

Corollary 4 demonstrates that (1)the recycling quantities of retailers and third-party recyclers in Model $I$ and Model $II$ of the closed-loop supply chain for the echelon recycling of power batteries embedded in the blockchain under the traceability mechanism are equal when they reach optimal profit. Additionally, the optimal recycling quantities in Model $I$ are higher than those in Model $II$ due to the smaller competition intensity of the recycling channel in Model $I$. (2) When the residual rate $\alpha$ of the SPBs into the stage of dismantling and utilization is large, the difference between the optimal recycling quantities of retailers and third-party recyclers in Mode $I$ and Mode $II$ increases with the increase of the spent power battery’s echelon utilization rate $\beta$, and conversely, the difference between the optimal recycling quantities decreases with the increase of $\beta$. This is because when $\alpha$ is larger, the manufacturer can get more recycled materials to produce power batteries. This is more profitable than using raw materials to produce power batteries, which prompts the manufacturer to produce more power batteries. As a result, the recycling quantity increases. The increase in recycling quantity is greater for Mode $I$, which has a lower competitive intensity of recycling subjects, than for Mode $II$. This leads to a greater difference between the recycling quantity of retailers and third-party recyclers in Mode $I$ and Mode $II$. On the contrary, when $\alpha$ is small, manufacturers will produce fewer power batteries, and the number of SPBs recycled will decrease, and the decrease in the number of recycled batteries in Mode $I$, where the intensity of competition among recycling entities is lower, is greater than that in Mode $II$. This results in a decrease in the difference in the number of recycled batteries recycled by retailers and third-party recyclers between Mode $I$ and Mode $II$. (3) As $\varphi$ and $\rho$ increase, the difference in recycling quantity between retailers and third-party recyclers decreases in both Mode $I$ and Mode $II$. Increases in $\varphi$ indicate a weakened ability for cost optimization, resulting in higher costs for laddering utilizers. This prompts a decrease in transfer prices, leading to reduced recycling prices for retailers and third-party recyclers. As a result, the quantity of recycling decreases. Increases in $\rho$ indicate higher manufacturing costs for power batteries, leading to a decrease in the number of batteries produced and recycled. The decrease in the number of recycled batteries is larger in Mode $I$ than in Mode $II$.

### Corollary 5

If $m > m_{2}$, then $Q^{{I^{*} }} < Q^{{II^{*} }}$; If $2n < m < m_{2}$ and $0 < F < F_{4}$, then $Q^{{I^{*} }} < Q^{{II^{*} }}$; If $2n < m < m_{2}$ and $F > F_{4}$, then $Q^{{I^{*} }} > Q^{{II^{*} }}$.

### Proof

See Online Appendix F.

Corollary 5 demonstrates that: (1) When the recycling channel’s competitive intensity is low and the recycling price sensitivity is high, the total recycled quantity of retired power batteries of Model $I$ is always smaller than that of Model $II$. This is because, although the sensitivity coefficient of competition in recycling channels is smaller, the sensitivity coefficient of recycling prices is larger, and each recycling participant is more sensitive to price changes, while the participation of echelon utilizers in recycling in Mode $II$ can offer higher recycling prices, which attracts more SPBs to the recycling market. (2) When the competition among recycling channels is high and the price sensitivity of recycling is low, and at the same time the level of cost optimization coefficient embedded in the blockchain technology is low, the total quantity of retired power batteries recycled for Model $I$ is smaller than that for Model $II$. Conversely, when the competition among recycling channels is higher and the price sensitivity of recycling is lower, and at the same time the level of cost optimization coefficient embedded in the blockchain technology is higher, the total quantity of retired batteries recycled for Model $I$ is greater than that for Model $II$. This is due to the fact that when the cost optimization factor of blockchain technology is at a low level, both echelon utilizers and manufacturers in the two modes tend to set lower transfer prices and recycling prices, and although the sensitivity coefficient of competition in recycling channels is larger, the lower sensitivity coefficient of recycling price makes the recycling quantity of SPBs in Mode $II$ higher than that in Mode $I$. However, if the cost optimization factor of blockchain technology is increased to a higher level, the echelon utilizers and manufacturers in both modes will increase the transfer price and recycling price accordingly, and this change will make the recycling quantity of SPBs in Mode $II$ lower than that in Mode $I$. The manufacturer must adjust the cost optimization coefficient based on the competitive intensity of the recycling channel, price sensitivity of recycling, and recycling mode. For instance, if the competition among recycling participants in the recycling channel is intense and consumers are moderately sensitive to the recycling price, the manufacturer should decrease the level of cost optimization coefficient for recycling mode $II$ and increase it for recycling mode $I$.

### Corollary 6

The profit relationship between the third-party recycler and the retailer under the two recycling models is as follows:

1. $$\pi_{r}^{{I^{*} }} > \pi_{r}^{{II^{*} }}$$, $$\pi_{t}^{{I^{*} }} > \pi_{t}^{{II^{*} }}$$;
2. If $1 > \alpha > \alpha_{1}$, then ${{\partial (\pi_{r}^{{I^{*} }} - \pi_{r}^{{II^{*} }} )} \mathord{\left/ {\vphantom {{\partial (\pi_{r}^{{I^{*} }} - \pi_{r}^{{II^{*} }} )} {\partial \beta }}} \right. \kern-0pt} {\partial \beta }} > 0$, ${{\partial (\pi_{t}^{{I^{*} }} - \pi_{t}^{{II^{*} }} )} \mathord{\left/ {\vphantom {{\partial (\pi_{t}^{{I^{*} }} - \pi_{t}^{{II^{*} }} )} {\partial \beta }}} \right. \kern-0pt} {\partial \beta }} > 0$; If $0 < \alpha < \alpha_{1}$, ${{\partial (\pi_{r}^{{I^{*} }} - \pi_{r}^{{II^{*} }} )} \mathord{\left/ {\vphantom {{\partial (\pi_{r}^{{I^{*} }} - \pi_{r}^{{II^{*} }} )} {\partial \beta }}} \right. \kern-0pt} {\partial \beta }} < 0$, ${{\partial (\pi_{t}^{{I^{*} }} - \pi_{t}^{{II^{*} }} )} \mathord{\left/ {\vphantom {{\partial (\pi_{t}^{{I^{*} }} - \pi_{t}^{{II^{*} }} )} {\partial \beta }}} \right. \kern-0pt} {\partial \beta }} < 0$;
3. $${{\partial (\pi_{r}^{{I^{*} }} - \pi_{r}^{{II^{*} }} )} \mathord{\left/ {\vphantom {{\partial (\pi_{r}^{{I^{*} }} - \pi_{r}^{{II^{*} }} )} {\partial \rho }}} \right. \kern-0pt} {\partial \rho }} < 0$$, $${{\partial (\pi_{t}^{{I^{*} }} - \pi_{t}^{{II^{*} }} )} \mathord{\left/ {\vphantom {{\partial (\pi_{t}^{{I^{*} }} - \pi_{t}^{{II^{*} }} )} {\partial \rho }}} \right. \kern-0pt} {\partial \rho }} < 0$$, $${{\partial (\pi_{r}^{{I^{*} }} - \pi_{r}^{{II^{*} }} )} \mathord{\left/ {\vphantom {{\partial (\pi_{r}^{{I^{*} }} - \pi_{r}^{{II^{*} }} )} {\partial \varphi }}} \right. \kern-0pt} {\partial \varphi }} < 0$$, $${{\partial (\pi_{t}^{{I^{*} }} - \pi_{t}^{{II^{*} }} )} \mathord{\left/ {\vphantom {{\partial (\pi_{t}^{{I^{*} }} - \pi_{t}^{{II^{*} }} )} {\partial \varphi }}} \right. \kern-0pt} {\partial \varphi }} < 0$$

### Proof:

See Online Appendix G.

Corollary 6 demonstrates that: (1) Given a recycling market size, both retailers and third-party recyclers in Model $I$ are more profitable than in Model $II$. This is due to the higher optimal quantity of recycling in Model $I$ compared to Model B, as well as the lower intensity of competition in the recycling channel in Model $I$. (2) The optimal profit difference between retailers and third-party recyclers in Model $I$ and Model $II$ increases with $\beta$ when $\alpha$ is larger; otherwise, it decreases with $\beta$. This is because manufacturers can obtain more recycled materials when the residual rate of power batteries entering the dismantling and utilization stage is higher. This is more profitable than using raw materials to produce power batteries, which enhances the manufacturer’s incentive to produce and recycle. As a result, the optimal profit increases successively. Mode $I$, which has a lower intensity of recycling competition, experiences a larger profit increase than Mode $II$. On the contrary, if the residual rate of power batteries during the dismantling and utilization stage is low, the manufacturer’s production of power batteries using recycled materials decreases. This leads to a decrease in marginal revenue, recycling incentives, and the number of recycling, resulting in a decrease in the manufacturer’s optimal profit. The decrease in profit is larger in Model $I$ than in Model $II$. (3) The difference in optimal profits between retailers and third-party recyclers in Mode $I$ and Model $II$ decreases as $\varphi$ and $\rho$ increase. This is because an increase in the optimization coefficient of the processing cost of the power battery’s echelon use, $\varphi$, weakens the ability to optimize the cost and increases the cost of echelon users. This drives the transfer price down, resulting in a decrease in optimal profits for both retailers and third-party recyclers. An increase in the optimization factor $\rho$ for the processing cost of power batteries using recycled materials results in an increase in the manufacturer’s production cost of power batteries. This, in turn, leads to an increase in the selling and recycling prices of power batteries. As a result, the optimal profit of retailers and third-party recyclers decreases. In Mode $I$, the decrease in profit is greater than that in Mode $II$.

### Corollary 7

If $2n < m < m_{2}$ and $F > F_{6}$, then $\pi_{c}^{{I^{*} }} > \pi_{c}^{{II^{*} }}$; If $2n < m < m_{2}$ and $0 < F < F_{6}$, then $\pi_{c}^{{I^{*} }} < \pi_{c}^{{II^{*} }}$; If $m > m_{2}$, then $\pi_{c}^{{I^{*} }} < \pi_{c}^{{II^{*} }}$.

### Proof

See Online Appendix H.

Corollary 7 demonstrates that: (1) the profit of Model $I$ is greater than that of Model $II$ when the competitive intensity of the recycling channel is high, recycling price sensitivity is low, and the level of cost optimization factor embedded in the blockchain technology is low. Conversely, when the competitive intensity of the recycling channel is high, recycling price sensitivity is low, and the level of cost optimization factor embedded in the blockchain technology is high, the profit of Model $I$ is less than that of Model $II$. This is due to the fact that when the cost optimization factor of blockchain technology is at a higher level, the cost of the echelon utilizer can be effectively reduced, which leads it to set a higher transfer price or recycling price, which effectively increases the amount of SPBs recycled, and despite the higher competitive sensitivity factor of the recycling channel, the profit of the echelon utilizer in Mode $II$ is lower than that in Mode $I$ due to the lower sensitive factor of the recycling price; On the contrary, when the cost optimization factor of the blockchain technology is low, the cost of the echelon utilizer will increase, thus setting a lower transfer price or recycling price, resulting in a lower recycling quantity of SPBs, and when the competitive sensitivity coefficient of the recycling channel is high, even if the sensitivity coefficient of the recycling price is low, the profit of the echelon utilizer in the more competitive Mode $II$ is greater than that of Mode $I$. (2) When the recycling channel’s competitive intensity is lower and the price sensitivity of recycling is higher, the echelon utilizer profit of Model $I$ is always less than that of Model $II$. This is because although the competitive sensitivity coefficient of the recycling channel is smaller, the price sensitivity coefficient is larger, and each recycling participant is more sensitive to price changes, while the participation of echelon utilizers in recycling in Mode $II$ is able to provide higher recycling prices and transfer prices, which attracts more SPBs to enter the recycling market, and thus the profits of echelon utilizers in Mode $II$ are higher than those in Mode $I$.

### Corollary 8

If $2n < m < m_{2}$ and $F > F_{5}$, then $\pi_{m}^{{I^{*} }} > \pi_{m}^{{II^{*} }}$; If $2n < m < m_{2}$ and $0 < F < F_{5}$, then $\pi_{m}^{{I^{*} }} < \pi_{m}^{{II^{*} }}$; If $m > m_{2}$, then $\pi_{m}^{{I^{*} }} < \pi_{m}^{{II^{*} }}$.

### Proof

See Online Appendix I.

Corollary 8 demonstrates that: (1) When the recycling channel’s competitive intensity is high, the recycling price’s sensitivity is low, and the blockchain technology’s cost optimization coefficient is low, the profit of Model $I$ is greater than that of Model $II$. Conversely, if the recycling channel’s competitive intensity is high, the recycling price’s sensitivity is low, and the blockchain technology’s cost optimization coefficient is high, the profit of Model $I$ is smaller than that of Model $II$. This is because when the cost optimization coefficient of blockchain technology is at a high level, the manufacturer’s cost can be effectively reduced, leading to the setting of a higher transfer price, and the amount of SPBs recycling will increase. Despite the high competitive sensitivity coefficient of recycling channels, the manufacturer’s profit in Mode $II$ is smaller than that in mode $I$ due to the low sensitive coefficient of recycling price. With a low cost optimization factor of blockchain technology, the manufacturer’s cost will be increased, which will cause it to set a lower transfer price, then the number of SPBs recycling will be reduced, and despite the high competitive sensitivity coefficient of the recycling channel, a sufficiently low sensitivity coefficient of the recycling price will lead to a larger manufacturer profit in Mode $II$ than in Mode $I$. (2) When the competition intensity in the recycling channel is lower and the sensitivity to recycling prices is higher, the manufacturer’s profit in Mode $I$ is always smaller than that in Mode $II$, regardless of the level of the cost optimization coefficient embedded in the blockchain technology. This is due to the fact that, according to Corollary 1, the manufacturer in Mode $I$ sells power batteries at a wholesale price and in quantities equal to that in Mode $II$. Therefore, the production cost of power batteries determines the profit size of the two models. Based on Corollary 3, the manufacturer’s price for recycling materials is higher in Model $I$ than in Model $II$. Therefore, the manufacturer’s marginal revenue from using recycled materials to produce power batteries in Model $I$ is lower than in Model $II$. Additionally, Corollary 5 states that the quantity of recycled materials in Model $I$ is lower than in Model $II$, resulting in lower profits for the manufacturer in Model $I$ compared to Model $II$.

Corollary 7 and Corollary 8 demonstrate that the manufacturer and echelon utilizer can both achieve maximum profit under the same conditions. This means that when the manufacturer earns the maximum profit, the echelon utilizer will also earn the maximum profit, resulting in a mutually beneficial outcome. This provides theoretical support for cooperation between the manufacturer and the echelon utilizer.

## Results and analysis

To analyze the impact of different parameters on optimal decision-making for manufacturers and echelon utilizers of blockchain-embedded power battery echelon recycling, this paper combines assumptions and draws on parameter settings from the literature 7 and 33. the optimal recycling quantity of SPBs and the optimal profits of manufacturers and secondary utilizers in different recycling modes are taken into account to be affected by the level of cost optimization coefficients embedded in blockchain technology, the level of consumer recycling prices, the degree of recycling price sensitivity, and the intensity of competition in recycling channels. Each inference’s accuracy is confirmed through MATLAB numerical simulation. The parameters are assigned as follows:$t = 0.25$, $\beta = 0.1$, $p_{s} = 2$, $c_{p} { = }0.6$, $p_{h} = 0.7$, $q = 0$, $m = 1.1$, $n = 0.3$, $k = 0.6$, $\alpha = 0.7$, $a = 10$, $b = 0.5$, $\varphi = 0.8$, $\rho = 0.8$, $c_{n} = 8$, $c_{r} = 7$, $\mu = 2$.

### Optimal total recovery quantity analysis

**Figure 2:**
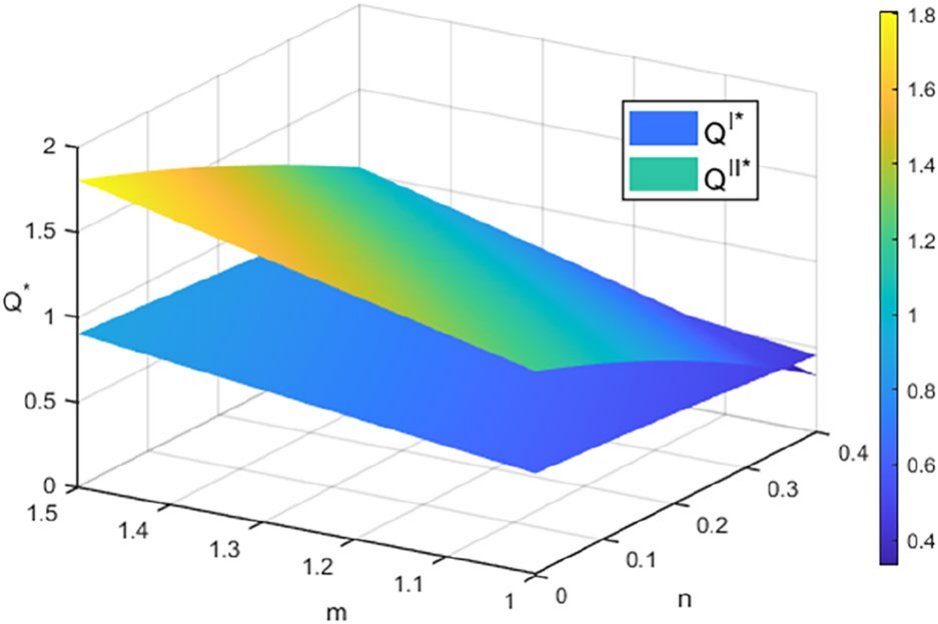
The effect of $m$ and $n$ on the number of recycles.

The recycling quantities of both recycling models increase as the sensitivity coefficient of recycling price $m$ increases and decrease as the sensitivity coefficient of recycling channel competition $n$ increases. Additionally, in Model $II$, the recycling quantities are more significantly affected by $n$. When $m$ is larger and $n$ is smaller, the recycling quantities in Model $I$ are consistently lower than those in Model $II$. Additionally, when the level of the cost optimization coefficient embedded in the blockchain technology is lower, indicating a higher cost optimization ability $\left( {F > F_{4} } \right)$, and when $m$ is smaller and $n$ is larger $\left( {2n < m < m_{2} } \right)$, the recycling quantity of power batteries in Mode $I$ is higher than that in Mode $II$, as shown in Fig. 2. The simulation results above align with the findings of Corollary 5. Compared to Mode $I$, Mode $II$ shows a greater change in the number of SPBs recycled due to increased competition among recycling channels and the participation of more recycling subjects.

**Figure 3:**
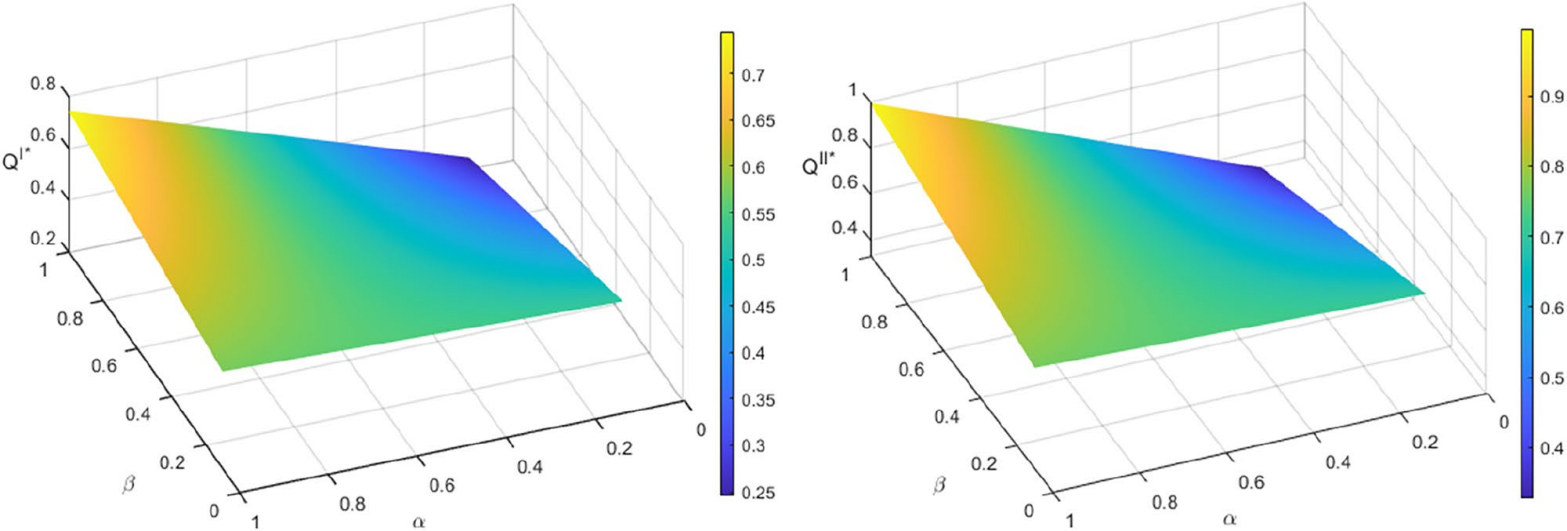
The influence of $\alpha$ and $\beta$ recycling quantity $Q^{I*}$ and $Q^{II*}$.

Figure 3 shows that the quantity of recycled materials increases with $\alpha$ in both recycling models. By increasing the transfer price, the manufacturer can obtain more recycled materials, which lowers the marginal cost of producing new batteries compared to using raw materials. This, in turn, allows for an increase in the recycled quantity of SPBs. The recycling quantity decreases as the SPBs’ echelon utilization rate $\beta$ increases, when the residual rate $\alpha$ of power batteries entering the dismantling and utilization stage is small. A smaller size of non-echelon utilization batteries results in higher costs for the manufacturer to dismantle and process them, extract recycled materials, and produce new power batteries. This lower profit margin reduces the manufacturer’s incentive to recycle non-echelon utilization power batteries. Corollaries 7 and 8 demonstrate that in this scenario, the recycling profit and motivation of the echelon utilizer will decrease. Additionally, an increase in $\beta$ will reduce the profit of both parties, leading the manufacturer and the echelon utilizer to opt for a lower transfer price, resulting in a reduction in recycling quantity. When $\alpha$ is larger, the quantity of recycling increases with the increase of $\beta$. This indicates that the manufacturer’s cost of disassembling and treating the non-echelon utilization batteries, extracting the recycled materials, and producing new power batteries is lower than the profit. As a result, the manufacturer’s profit and incentive to recycle the non-echelon utilization power batteries increase. It is evident from corollaries 7 and 8 that the recycling profit and incentive of the echelon utilizer increase in this scenario. Additionally, an increase in $\beta$ leads to higher profits for both parties. Both the manufacturer and echelon utilizer opt to raise the transfer price to boost the recycling quantity.

**Figure 4:**
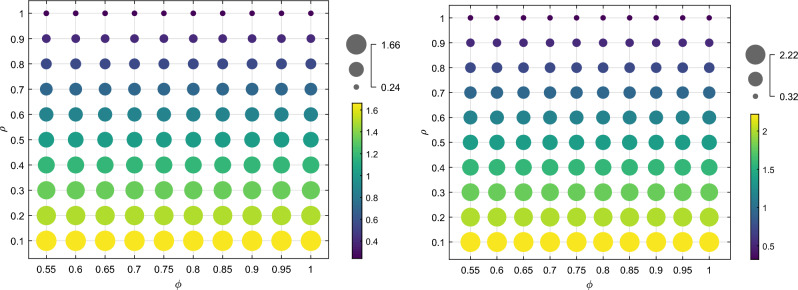
The influence of $\alpha$ and $\beta$ recycling quantity $Q^{I*}$ and $Q^{II*}$.

Figure 4 shows that as the color of the phase changes from the lower left to the upper right of the bubble map, and the area decreases, the cost optimization ability decreases. This is due to the increase in the cost optimization coefficient $\varphi$ of the echelon utilizer and the cost optimization coefficient $\rho$ of the manufacturer embedded in the blockchain technology under the traceability mechanism. The recycling quantity of the power battery under the two recycling modes is on a downward trend. The increase in the cost optimization coefficient $\varphi$ for the echelon utilizer results in a rise in marginal cost and a decrease in transfer and recycling prices. This leads to a decrease in the recycling price for both the retailer and third-party recycler, ultimately reducing losses by decreasing the recycling quantity. An increase in $\rho$ results in an increase in the marginal cost, causing the manufacturer to reduce losses by lowering the transfer price. This, in turn, leads to a rise in the marginal cost of the echelon utilizer and a decrease in the recycling price for retailers and third-party recyclers, ultimately resulting in a decrease in the recycling quantity. Furthermore, the change in recycling quantity with respect to $\rho$ is more significant than the change with respect to $\varphi$, when a certain amount of $\varphi$ is held constant and $\rho$ remains unchanged. This is due to the manufacturer’s dominant position in the recycling process and their role as the final link in the echelon recycling of SPBs. The recycling cost set by the manufacturer determines the price of the entire closed-loop supply chain. If the dismantling and utilization of a product cannot be significantly improved to optimize costs, and there is an upper limit on the marginal gain, then the amount of recycling will be determined by the marginal gain. In this scenario, even if blockchain technology is embedded in the cost of recycling for echelon recyclers, it may not result in a significant change in the amount of recycling.

### Comparison of optimal profitability and sensitivity analysis for manufacturers and echelon utilizers

**Figure 5:**
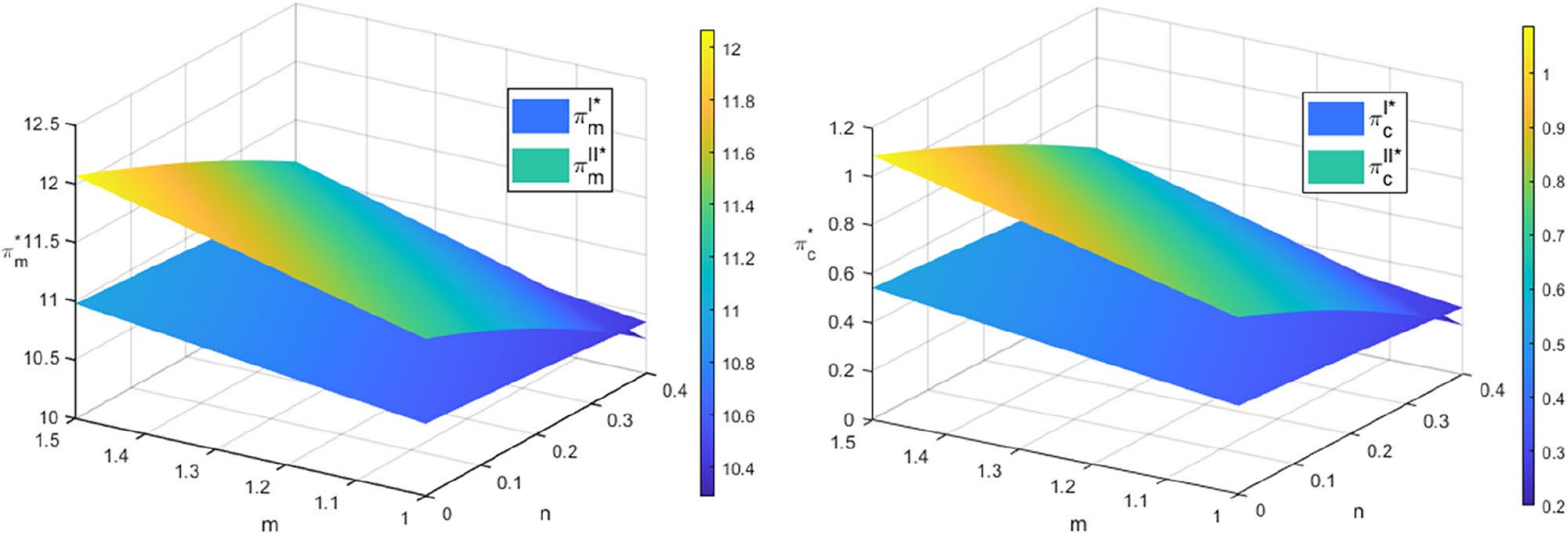
The impact of $m$ and $n$ on the profits of manufacturers and echelon users.

As shown in Fig. 5, the manufacturer’s profit increases with an increase in $m$ and decreases with an increase in $n$ in both recycling modes. The echelon utilizer also changes synchronously with the manufacturer’s profit, but the change amplitude is more significant in mode $II$ than in mode $I$. The simulation results demonstrate that the manufacturer is responsible solely for disassembling and extracting recycled materials from SPBs that cannot be used in a echelon manner to produce new power batteries, and the larger the amount of recycled SPBs, the higher the profit of echelon utilizers, and the more the manufacturer saves the material cost, and the profit is synchronously increased, which is consistent with the theoretical results in the literature^6,37^. The recycling price increases as the sensitivity coefficient of recycling channel competition grows, leading to greater competition in the spent power battery recycling market and decreased profits for manufacturers and echelon utilizers, while the intensity of competition among recycling participants is greater in Model $II$ with more recycling channels, and the magnitude of the change in manufacturers’ and echelon utilizers’ profits in response to recycling channel competition is more significant.

In Fig. 5, the intersection of the two planes indicates that when the competitive intensity of the recycling channel is low and the recycling price sensitivity is high $\left( {m > m_{2} } \right)$, Manufacturers and ladder utilizers in Model $I$ are less profitable than those in Model $II$. In addition, when the cost optimization ability is high $\left( {F > F_{5} } \right)$ and the competitive intensity of the recycling channel is high while the recycling price sensitivity is low $\left( {2n < m < m_{2} } \right)$, Manufacturers and ladder utilizers in Model $I$ are more profitable than those in Model $II$. This finding is consistent with Corollaries 7 and 8. The magnitude of changes in manufacturer and echelon utilizer profits with the intensity of recycling competition in Model $II$ is more pronounced compared to Model $I$. The reason for this is that the rise in recycling participants in Model $II$ intensifies competition in the channel, thereby making the impact of competition on the profits of manufacturers and echelon utilizers more pronounced.

**Figure 6:**
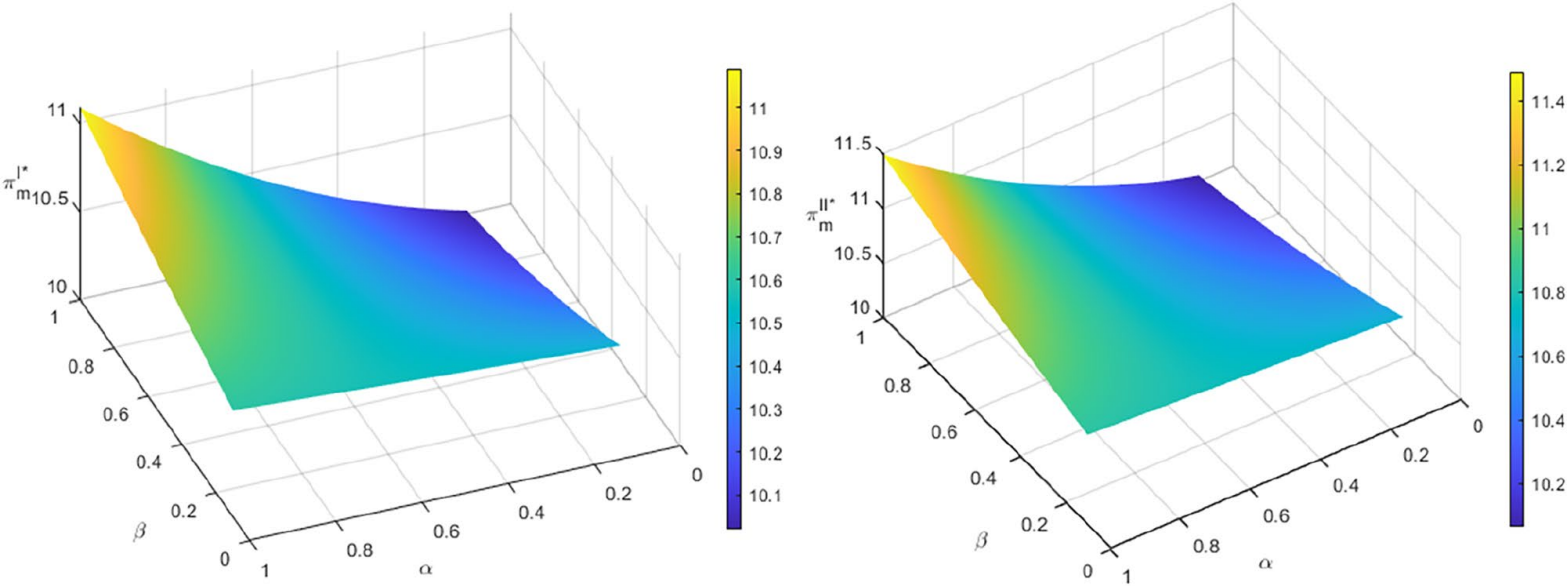
The impact of $\alpha$ and $\beta$ on the profits of manufacturers $\pi_{m}^{I*}$ and $\pi_{m}^{II*}$.

**Figure 7:**
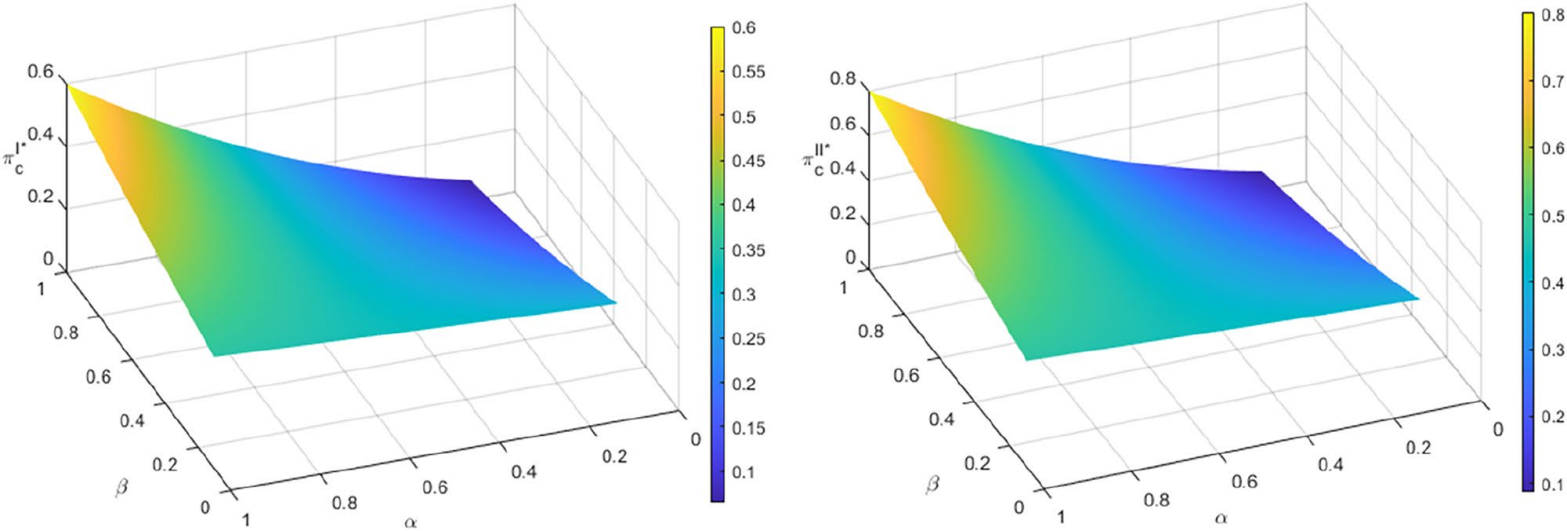
The impact of $\alpha$ and $\beta$ on the profits of echelon users $\pi_{c}^{I*}$ and $\pi_{c}^{II*}$.

Figures 6 and 7 demonstrate that increasing both $\beta$ and $\alpha$ results in an upward trend in profits for manufacturers and echelon utilizers in the two recycling modes. This may be attributed to the embedding of blockchain technology in the process of echelon recycling and utilization of power batteries in the standardization of the spent battery market. The information on remaining capacity is now more transparent, which has led to increased transaction activity among market participants. This has also enhanced trust, and the profits obtained by manufacturers as a terminal link and key link echelon utilizer have grown synchronously with both $\beta$ and $\alpha$. However, when $\alpha$ is small, the profits of manufacturers and echelon utilizers decrease as $\beta$ increases. According to Assumption 4, recycled materials extracted from non-echelon-utilized batteries can satisfy the production of new batteries at a lower cost than producing power batteries using new materials^6,37^. However, the residual rate of recycled materials at the dismantling and utilization stage is low, and manufacturers can only support a limited amount of recycled materials for new battery production, resulting in the limited reduction of marginal production cost. Moreover, the current technical level of recovery of recycled materials by manufacturers needs to be further improved, and some of the recyclable materials have entered the final form and have not been used in the production of new batteries so that the production cost of manufacturers cannot be significantly reduced by using recycled materials. When $\alpha$ is larger, the profits of manufacturers and echelon utilizers increase with the increase of $\beta$. This indicates that the echelon utilizers have achieved a higher level of value transformation of SPBs before entering the dismantling and utilization stage. After entering the dismantling and utilization stage, the manufacturers have a higher level of technology to extract recyclable materials for the production of new power batteries. This leads to a reduction in the marginal cost of production, achieving the ideal state of the closed-loop supply chain of SPBs for echelon utilization under the mechanism of traceability.

**Figure 8:**
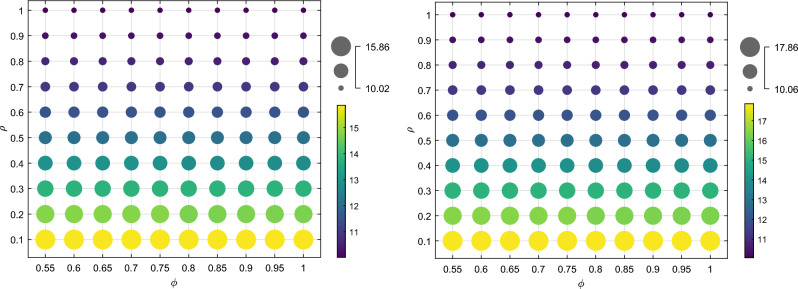
The impact of $\varphi$ and $\rho$ on the profits of manufacturers $\pi_{m}^{{I{*}}}$ and $\pi_{m}^{{II{*}}}$.

**Figure 9:**
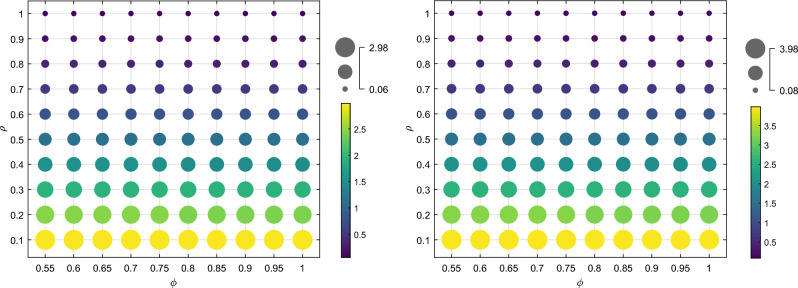
The impact of $\varphi$ and $\rho$ on the profits of echelon users $\pi_{c}^{I*}$ and $\pi_{c}^{II*}$.

As shown in Figs. 8 and 9, the color of the lower left to upper right phase of the bubble diagram darkens gradually, and the area decreases, indicating that the profits of both the manufacturer and the echelon utilizer decrease in both recycling modes as the cost optimization coefficient $\varphi$ of the echelon utilizer and the cost optimization coefficient $\rho$ of the manufacturer increase with the embedding of blockchain technology under the traceability mechanism. From Sect. 6.1, it is evident that the recycling quantity of SPBs decreases as $\varphi$ and $\rho$ increase. This results in a reduction of the proportion of recycled materials used in the production of new batteries by the manufacturer. As a consequence, the marginal production cost increases, and the profit decreases. The optimal profit conditions of the echelon utilizer and the manufacturer remain the same, as shown in corollary 7 and corollary 8. The profit of the echelon utilizer decreases accordingly. In addition, the profit of manufacturers and echelon utilizers is more significant when $\rho$ changes while $\varphi$ remains constant, compared to when $\varphi$ changes while $\rho$ remains constant. This is due to the dominant position of the manufacturer in the recycling process and their involvement in the final stage of echelon recycling of SPBs. The price of the entire closed-loop supply chain is determined by its recycling cost. If the cost optimization ability of dismantling and utilization is not significantly improved, there will be an upper boundary of marginal revenue. This is a key factor in determining the recycling quantity and profit. Even if the blockchain is embedded in the recycling cost of echelon utilizers with a large range of changes, it cannot bring about a significant change in profit.

### Blockchain technology embedding and consumer traceability information preference analysis

**Figure 10:**
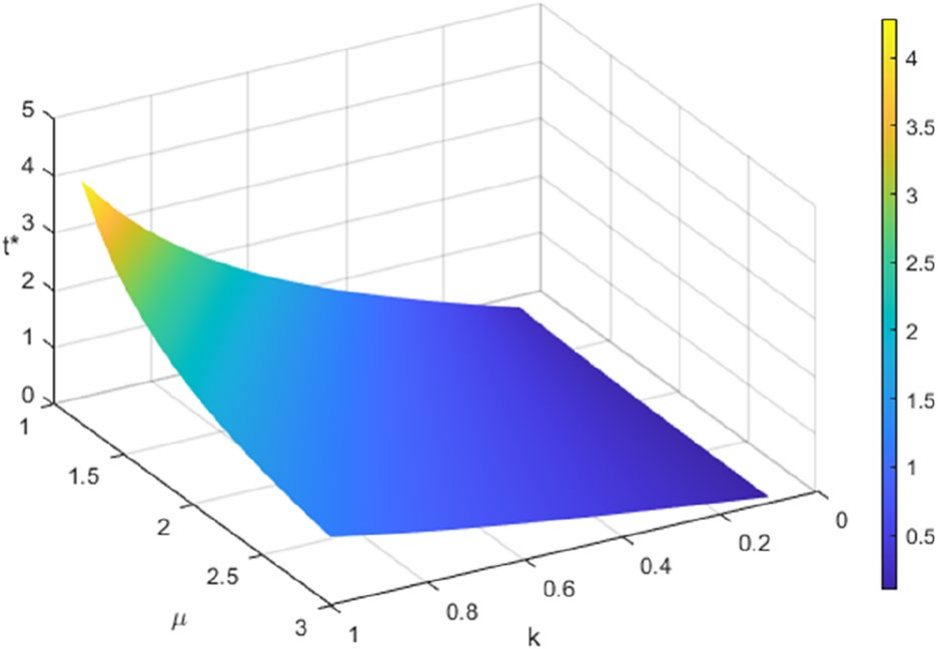
The influence of $\mu$ and $k$ on the optimal blockchain technology embedding level.

**Figure 11:**
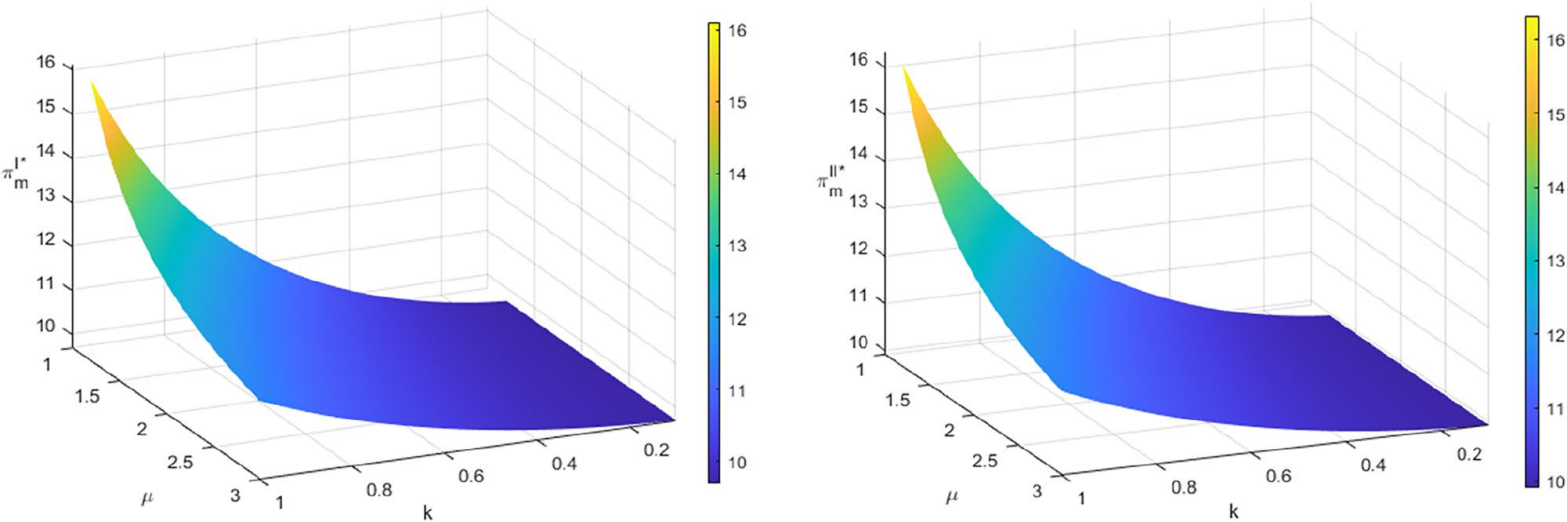
The impact of $\mu$ and $k$ on the profits of manufacturers $\pi_{m}^{{I{*}}}$ and $\pi_{m}^{{II{*}}}$.

According to Proposition 1, the optimal level of blockchain technology embedding is equal under both recycling modes, expressed as $$t^{*}$$$$\left( {t^{*} = t^{{I{*}}} = t^{{II{*}}} } \right)$$. Figures 10 and 11 show that the level of blockchain technology embedding and the manufacturer’s profit increase with the increase of the coefficient of consumer preference for traceability information $$k$$ and decrease with the increase of the coefficient of investment cost of blockchain technology $$\mu$$. This simulation result demonstrates that as an emerging market, consumers have a strong demand for clear and traceable identification of the remaining capacity of power batteries that can be utilized in an echelon manner. The embedding of blockchain technology can help establish trust in transactions between consumers, reduce the cost of market transactions, expand market demand, and increase the amount of SPBs recycled. However, the manufacturer’s increased input cost for blockchain technology leads to a rise in marginal cost. While it may increase the recycling volume of decommissioned power batteries and expand the market scale, it remains uncertain whether it can generate profits within a short payback period and cover the upfront investment cost of blockchain technology. The manufacturer must consider various factors, such as the intensity of competition in the recycling channel, the sensitivity of the recycling price, the residual rate of the echelon recycling, and other parameters to make an optimal decision.

## Conclusion

### Research conclusion

This paper proposes two modes of blockchain technology embedded in power battery echelon recycling under a traceability mechanism. It considers the impact of blockchain technology embedded in the power battery closed-loop supply chain under the two modes. The main conclusions are as follows:

1. In the forward supply chain, different recycling modes in the reverse supply chain do not affect the manufacturer’s optimal wholesale price, the level of blockchain technology embedding, the retailer’s optimal retail price, and the consumer’s optimal demand quantity. The decision to sell in the forward supply chain is independent of the decision to recycle in the reverse supply chain. The sales and recycling of power batteries are two distinct and separate businesses.
2. Model $I$ has higher transfer prices for both manufacturers and echelon utilizers compared to Model $II$. The level of blockchain technology embedding decreases as the investment cost coefficients increase but increases with the increase of the preference coefficients for traceability information. Additionally, retailers and third-party recyclers in Model $I$ have higher recycling quantities and profits than those in Model $II$.
3. The quantity of SPBs that should be recycled optimally is influenced by the intensity of recycling competition, sensitivity to recycling prices, and the level of blockchain technology embedding. When recycling competition intensity is low and recycling price sensitivity is high, the optimal recycling mode is Mode $II$. When recycling competition intensity is high, recycling price sensitivity is low, and the cost optimization coefficient embedded in blockchain technology is high, the optimal recycling mode is Mode $I$. When recycling competition is intense but recycling prices are not sensitive and the cost optimization coefficient embedded in blockchain technology is low, the optimal recycling mode is Mode $II$. The quantity of power battery recycling increases with the increase of recycling price sensitivity coefficient and decreases with the increase of recycling competition intensity. The recycling quantity is affected by the residual rate of power batteries entering the dismantling and utilization stage and the echelon utilization rate of SPBs. Specifically, when the residual rate is small, the recycling quantity decreases as the echelon utilization rate increases. Conversely, when the residual rate is large, the recycling quantity increases as the echelon utilization rate increases.
4. The intensity of recycling competition, recycling price sensitivity, and the level of blockchain technology embedding affect both manufacturers and echelon utilizers. When recycling competition intensity is low and recycling price sensitivity is high, the optimal recycling mode is mode B. However, when recycling competition intensity is high and recycling price sensitivity is low, and the level of cost optimization coefficient embedded in the blockchain technology is low, the optimal recycling modes for the manufacturer and the echelon utilizer are mode A. If the cost optimization coefficient embedded in the blockchain technology is high, the optimal recycling mode for the manufacturer and the echelon utilizer is mode B. The profit of both the manufacturer and the echelon utilizer is affected by the price sensitivity coefficient of spent power battery recycling and the residual rate of power batteries entering the dismantling and utilization stage. Additionally, it decreases as the competitive intensity of power battery recycling for echelon utilization, cost optimization coefficient of the echelon utilizer, and cost optimization coefficient of the manufacturer increase. When the residual rate of power batteries entering the dismantling and utilization stage is low, the profits of manufacturers and echelon utilizers decrease as the echelon utilization rate of the SPBs increases. When the residual rate of power batteries entering the dismantling and utilization stage is high, the profits of manufacturers and echelon utilizers increase as the echelon utilization rate of the retired power batteries increases.

### Managerial implications

Based on the above research conclusions, the following management insights can be obtained:

1. Embed blockchain technology in the supply chain of secondary recycling and utilization of power batteries under the traceability mechanism. Echelon utilizers should base their recycling mode decisions on the intensity of recycling competition, sensitivity to recycling prices, and the level of cost optimization coefficient. When the optimal profit scale of manufacturers and echelon utilizers grows synchronously under the two modes, manufacturers should establish a long-term cooperation mechanism with secondary utilizers. This includes building an information traceability chain that covers logistics, transportation, production, and sales. It is important to promote the construction of a traceability mechanism embedded in blockchain technology and optimize the content and transparency of information disclosure of battery residual capacity. This will increase the importance of hidden value and trust of market transactions between manufacturers and echelon utilizers, and help to realize the operability and popularity of the extended producer responsibility system.
2. In the current context of recycled material extraction and blockchain technology, it is important to note that the echelon utilization rate of power batteries should not be maximized without consideration of residual rates during the dismantling and utilization stages. If the residual rate is too small, the profit obtained from producing new power batteries with recycled materials may not cover the cost of dismantling power batteries that cannot be utilized in an echelon extraction of recycled materials, the manufacturer’s recovery of the marginal cost of power batteries will increase. Currently, higher recycling rates result in greater financial losses for both the manufacturer and the echelon recycler. To address this issue, manufacturers should focus on improving the level of dismantling and utilization, as well as implementing blockchain technology to enhance the efficiency of the dismantling and recycling processes.
3. The cost-effectiveness of blockchain technology is a crucial factor in deciding to embed the technology in the echelon recycling utilization of power batteries. The cost-effectiveness includes the size of the investment cost of the blockchain and the degree of utility value added by the consumer’s traceability preference, and an increase in the investment cost coefficient of the blockchain technology will lead to a decrease in the level of embedding of the blockchain technology and a decrease in the manufacturer’s profit under the two recycling modes, The recycling of power batteries may be hindered if the investment cost coefficient of the blockchain is too low. This could result in the manufacturer’s utility value added from the consumer’s traceability preference being lower than the cost effect of the embedding of the blockchain technology. In this case, the government should strengthen the manufacturer’s implementation of the extended producer responsibility system and provide subsidies. Additionally, the government should unite digital service platforms, financial service institutions, and the main enterprises of the power battery manufacturing chain to actively promote the coalition of SPBs capacity for digital labeling and standard construction. This will reduce the manufacturer’s investment in the cost of the blockchain. Moreover, to promote the embedding of its entire chain of the blockchain, and the effective transmission of the blockchain credit, which will create a higher value, and effectively improve the rights and interests of consumers, expand the overall size of the market for echelon recycling of the overall scale and completeness of the recycling market, to increase the profitability of the supply chain system.

Currently, the market for recycling power batteries is still in its growth stage. Other recycling methods include direct manufacturer participation in recycling and joint recycling among manufacturers. This paper focuses on the recycling of SPBs and explores two typical recycling modes: one in which SPB users participate in recycling and one in which they do not. The degree of marking the capacity of SPBs and consumer trust in the market are key factors that influence the recycling of SPBs. Thus, this study analyzes the impact of blockchain technology on the traceability mechanism, specifically on the power battery recycling volume and the profit of the recycling participant in the two modes mentioned above. The government’s behavior in the supply chain decision-making of the recycling participant is not taken into account. Additionally, the demand for power batteries is modeled as a stochastic function, and this paper simplifies it linearly without affecting the main inference. Future research will explore the impact of blockchain technology on the carbon quota mechanism and its effect on the power battery recycling volume and the profit of the recycling participant.

### Supplementary Information

Supplementary Information.

## Data Availability

The original contributions presented in the study are included in the article, further inquiries can be directed to the corresponding author.
