# Supplementary material for: Blockchain technology embedded in the power battery for echelon recycling selection under the mechanism of traceability
Source: Sci Rep. 2024 Jul 2;14:15069. doi: 10.1038/s41598-024-65748-0 (PMC11219883; doi:10.1038/s41598-024-65748-0)
Supplement: Supplementary file 1 — Supplementary Information. [file 41598_2024_65748_MOESM1_ESM.docx]

**Appendix**

***Appendix A***

Using the inverse solution method, compute the retailer's $p$ and $p_{r}$. The retailer's profit function $\pi_{r}^{I}$ has a Hessian matrix $H_{r}=\left[\begin{matrix} -2b & 0 \\ 0 & -2m \end{matrix}\right]$ with respect to $p$ and $p_{r}$. Since $b>0$，$m>0$， it can be shown that $\pi_{r}^{I}$ has a negatively determined Hessian matrix with respect to $p$ and $p_{r}$, and thus $\pi_{r}^{I}$ is a joint concave function with respect to $p$ and $p_{r}$. Letting $\partial\pi_{r}^{I}/\partial p=0$ and $\partial\pi_{r}^{I}/\partial p_{r}=0$， the retailer's reaction function with respect to $p$ and $p_{r}$: $p=\left(wb+kt+a\right)/2b$, $p_{r}=\left(p_{b}m+np_{t}-q\right)/2m$. Then compute the third-party recycler's recycling price $p_{t}$. The Hessian matrix $H_{t}=-2m$of the third-party recycler's profit function $\pi_{t}^{I}$ shows that $\pi_{t}^{I}$ is negatively definite concerning the Hessian matrix of $p_{t}$, and hence $\pi_{t}^{I}$ is a concave function with respect to $p_{t}$. Let $\partial\pi_{t}^{I}/\partial p_{t}=0$, the reaction function of $p_{t}$ can be obtained: $p_{t}=\left(p_{b}m+np_{r}-q\right)/2m$. Solve for the expressions of associative $p_{t}$ and $p_{r}$: $p_{r}\text{=}\left(p_{b}m-q\right)/\left(2m-n\right)$, $p_{t}\text{=}\left(p_{b}m-q\right)/\left(2m-n\right)$. Substituting variables $p$, $p_{t}$ and $p_{r}$ into the profit function $\pi_{c}^{I}$ of the echelon utilizer, we obtain a Hessian matrix $H_{c}=\left[4m\left(n-m\right)/\left(2m-n\right)\right]$ about $p_{b}$. Since $m>n$, $\pi_{c}^{I}$ has a negative definite Hessian matrix about $p_{b}$. Thus, $\pi_{c}^{I}$ is a concave function about $p_{b}$. Let $\partial\pi_{c}^{I}/\partial p_{b}=0$, we get the reaction function of $p_{b}$ : .

The manufacturer's profit function $\pi_{m}^{I}$ is obtained by substituting the reaction functions of $p$, $p_{t}$, $p_{r}$ and $p_{b}$. The Hessian matrix of $\pi_{m}^{I}$ with respect to $w$, $p_{m}$ and $t$ is $H_{m}=\left[\begin{matrix} -b & 0 & k/2 \\ 0 & 2m\left(1+\left(\alpha-1\right)\beta\right)^{2}\left(n-m\right)/\left(2m-n\right) & 0 \\ k/2 & 0 & -\mu\end{matrix}\right]$. When $\mu>k^{2}/4b$, $H_{m}$ is negatively definite. Therefore, $\pi_{m}^{I}$ is a joint concave function with respect to $w$, $p_{m}$ and $t$. To obtain the optimal solution of $\pi_{m}^{I}$ with respect to $w$, $p_{m}$ and $t$, let $\partial\pi_{m}^{I}/\partial w=0$, $\partial\pi_{m}^{I}/\partial p_{m}=0$ and $\partial\pi_{m}^{I}/\partial t=0$. Finally, substituting $w^{I^{*}}$,$p_{m}^{I^{*}}$ and $t^{I^{*}}$ into the reaction functions of $p$, $p_{t}$, $p_{r}$ and $p_{b}$ yields $p^{I^{*}}\text{=}\left(\left(c_{n}b+3a\right)\mu-c_{n}k^{2}\right)/\left(4\mu b-k^{2}\right)$ ,$p_{t}^{I^{*}}=\left(F m^{2}-Fnm-7qm+4nq\right)/\left(8m^{2}-12mn+4n^{2}\right)$, $p_{r}^{I^{*}}=\left(F m^{2}-Fnm-7qm+4nq\right)/\left(8m^{2}-12mn+4n^{2}\right)$, $p_{b}^{I^{*}}=\left(\left(m-n\right)F-3q\right)/\left(4m-4n\right)$.

***Appendix B***

The expressions for $w^{I^{*}}$, $w^{II^{*}}$, $\text{t}^{I^{*}}$, $\text{t}^{II^{*}}$, $\text{p}^{I^{*}}$and $\text{p}^{II^{*}}$ demonstrate that $w^{I^{*}}=w^{II^{*}}$,$t^{I^{*}}=t^{II^{*}}$and $p^{I^{*}}=p^{II^{*}}$. Additionally, $D^{I^{*}}=D^{II^{*}}\text{=}\left(b\mu\left(-bc_{n}+a\right)\right)/\left(4b\mu-k^{2}\right)$ can be obtained through calculation. Therefore, the proof is complete.

***Appendix C***

$\partial\pi_{m}^{I*}/\partial\alpha=\left(-F\left(m-n\right)-q\right)\left(c_{r}\rho-c_{n}\right)\beta m/\left(4m-2n\right)$, Given that $F\left(m-n\right)>0$, $q>0$ and $c_{r}\rho-c_{n}<0$, it follows that $-F\left(m-n\right)-q<0$ and $c_{r}\rho-c_{n}<0$, which implies that $\partial\pi_{m}^{I*}/\partial\alpha>0$.$\partial\pi_{m}^{II*}/\partial\alpha=\left(4m+n\right)\left(m-2n\right)\left(-F-q\right)\left(c_{r}\rho-c_{n}\right)\beta/\left(8m-4n\right)$, it is clear that $\partial\pi_{m}^{I*}/\partial\mu=\partial\pi_{m}^{II*}/\partial\mu<0$. Furthermore, $\partial\pi_{m}^{I*}/\partial\rho=\left(1+\left(\alpha-1\right)\beta\right)\left(-F\left(m-n\right)\right)mc_{r}/\left(4m-2n\right)$, since $1-\left(1-\alpha\right)\beta>0$ and $F\left(n-m\right)<0$, it follows that $\partial\pi_{m}^{I*}/\partial\rho<0$. $\partial\pi_{m}^{II*}/\partial\rho=\left(1+\left(\alpha-1\right)\beta\right)\left(-F\left(m-2n\right)-q\right)\left(4m+n\right)c_{r}/\left(8m-4n\right)$, since $1+\left(\alpha-1\right)\beta>0$and$-F\left(m-2n\right)-q<0$, we can conclude that $\partial\pi_{m}^{II*}/\partial\rho<0$. Lastly, $\partial\pi_{m}^{I*}/\partial k\text{=}\partial\pi_{m}^{II*}/\partial k\text{=}k\mu\left(-c_{n}b+a\right)^{2}/\left(4\mu b-k^{2}\right)^{2}$, we can see that $\partial\pi_{m}^{I*}/\partial k\text{=}\partial\pi_{m}^{II*}/\partial k>0$.

***Appendix D***

$p_{m}^{I^{*}}-p_{m}^{II^{*}}\text{=}nq/2\left(m-n\right)\left(1+\left(\alpha-1\right)\beta\right)\left(m-2n\right)$, from proposition 2, if $m>2n$, then $p_{m}^{I^{*}}>p_{m}^{II^{*}}$. To ensure that the solutions for $p_{b}^{I^{*}}$ and $p_{b}^{II^{*}}$ are meaningful, we assume $F>3q/\left(m-2n\right)$. $p_{b}^{I^{*}}-p_{b}^{II^{*}}=\left(\left(m-n\right)F-3q\right)\left(\left(m-2n\right)F-3q\right)/16\left(m-n\right)\left(m-2n\right)$, it is evident that $p_{b}^{I^{*}}>p_{b}^{II^{*}}$.

The equivalence relation between $p_{r}^{I^{*}}=p_{t}^{I^{*}}$ and $p_{r}^{II^{*}}=p_{t}^{II^{*}}$ can be directly proven from the expression. Here is a proof of the inequality relation: To ensure that $p_{r}^{I^{*}}$ and $p_{r}^{II^{*}}$ are meaningful, assume $p_{r}^{I^{*}}>0$ and $p_{r}^{II^{*}}>0$, which implies $F>q(7m-5n)/(m+n)(m-2n)$ and $F>q(7m-4n)/(m-n)m$, and let $F_{1}=q(7m-5n)/(m+n)(m-2n)$, $F_{2}=q(7m-4n)/(m-n)m$. $p_{r}^{I*}-p_{r}^{II*}=-n\left(F\left(m-n\right)\left(m-2n\right)-3q\left(2m-n\right)\right)/4\left(2m-n\right)\left(m-n\right)\left(m-2n\right)$, when $p_{r}^{I*}=p_{r}^{II*}$, it follows that$F=q(6m-3n)/(m-n)(m-2n)$, where $F_{3}=q(6m-3n)/(m-n)(m-2n)$. When $2n<m<m_{1}$, it follows that $F_{3}>F_{1}>F_{2}$, and if $F>F_{3}$, then $p_{r}^{I^{*}}<p_{r}^{II^{*}}$, where $m_{1}=(15+\sqrt{193})/\left(2n\right)$; if $F_{1}<F<F_{3}$, then $p_{r}^{I^{*}}>p_{r}^{II^{*}}$. When $m>m_{1}$, it gives $F_{3}<F_{1}<F_{2}$, and if $F>F_{2}$, then $p_{r}^{I^{*}}<p_{r}^{II^{*}}$; if $F_{1}<F<F_{2}$, then $p_{r}^{I^{*}}>p_{r}^{II^{*}}$.

***Appendix E***

(1)Based on the optimal decisions in Model $I$ and Model $II$, we can conclude that $Q_{t}^{I*}=Q_{r}^{I*}\text{=}m\left(\left(m-n\right)F+q\right)/\left(8m-4n\right)$， $Q_{t}^{II*}=Q_{r}^{II*}\text{=}\left(\left(m-2n\right)F+q\right)m/\left(8m-4n\right)$. From this, we can also deduce that , since and $m>2n$, which leads to $Q_{r}^{I^{*}}-Q_{r}^{II^{*}}=Q_{t}^{I^{*}}-Q_{t}^{II^{*}}>0$. (2), when $1>\alpha>\alpha_{1}$, $\partial(Q_{r}^{I^{*}}-Q_{r}^{II^{*}})/\partial\beta=\partial(Q_{t}^{I^{*}}-Q_{t}^{II^{*}})/\partial\beta>0$, conversely, when $0<\alpha<\alpha_{1}$, $\partial(Q_{r}^{I^{*}}-Q_{r}^{II^{*}})/\partial\beta=\partial(Q_{t}^{I^{*}}-Q_{t}^{II^{*}})/\partial\beta<0$,where ; (3) $\partial(Q_{r}^{I^{*}}-Q_{r}^{II^{*}})/\partial\rho=\partial(Q_{t}^{I^{*}}-Q_{t}^{II^{*}})/\partial\rho\text{=}-mnc_{r}\left(\beta\alpha-\beta+1\right)/\left(8m-4n\right)$, it is evident that $\partial(Q_{r}^{I^{*}}-Q_{r}^{II^{*}})/\partial\rho=\partial(Q_{t}^{I^{*}}-Q_{t}^{II^{*}})/\partial\rho<0$and , and it is also clear that .

***Appendix F***

$Q^{I^{*}}-Q^{II^{*}}=\left(\left(-2\left(m-5n/4\right)^{2}+41n^{2}/8\right)F-\left(n+2m\right)q\right)/\left(8m-4n\right)$， since $m>2n$, we can conclude that $8m-4n>0$. Therefore, the positive and negative signs of the numerator determine the magnitude of $Q^{I^{*}}$ and $Q^{II^{*}}$. The solution for $Q^{I^{*}}-Q^{II^{*}}=0$ with respect to $F$ is $F_{4}\text{=}q\left(2m+n\right)/\left(-2\left(m-5n/4\right)^{2}+41n^{2}/8\right)$. The solutions for $-2\left(m-5n/4\right)^{2}+41n^{2}/8=0$ are $m_{2}=\left(5+\sqrt{41}\right)/\left(4n\right)$ and $m_{3}=\left(5-\sqrt{41}\right)/\left(4n\right)$ respectively, and $m_{3}$ is omitted since $m>0$. When $m>m_{2}$, the coefficient of $F$ is less than zero and $F_{4}<0$. Since $F>0$, $Q^{I^{*}}<Q^{II^{*}}$. When $2n<m<m_{2}$, the coefficient of $F$ is greater than zero and $F_{4}>0$. If $0<F<F_{4}$, then $Q^{I^{*}}<Q^{II^{*}}$, and if $F>F_{4}$, then $Q^{I^{*}}>Q^{II^{*}}$.

***Appendix G***

(1) $\pi_{t}^{I^{*}}-\pi_{t}^{II^{*}}=m^{2}\left(\left(m-n\right)F+q\right)^{2}\left(\left(m-2n\right)F+q\right)^{2}/256\left(2m-n\right)^{4}$, it is clear that $\pi_{t}^{I^{*}}>\pi_{t}^{II^{*}}$. $\pi_{r}^{I^{*}}-\pi_{r}^{II^{*}}=Fmn\left(F\left(m-3n/2\right)+q\right)/8\left(2m-n\right)^{2}$, since $m-3n/2>0$, $\pi_{r}^{I^{*}}>\pi_{r}^{II^{*}}$.(2) , , where $I=3\left(m-n\right)\left(m-2n\right)Fqn/2>0$, it is clear that when $\alpha>\alpha_{1}$, it follows that $\partial(\pi_{r}^{I^{*}}-\pi_{r}^{II^{*}})/\partial\beta>0$ and $\partial(\pi_{t}^{I^{*}}-\pi_{t}^{II^{*}})/\partial\beta>0$; if $\alpha<\alpha_{1}$, then $\partial(\pi_{r}^{I^{*}}-\pi_{r}^{II^{*}})/\partial\beta<0$ and $\partial(\pi_{t}^{I^{*}}-\pi_{t}^{II^{*}})/\partial\beta<0$. (3) $\partial(\pi_{r}^{I^{*}}-\pi_{r}^{II^{*}})/\partial\rho=-nmc_{r}\left(1+\left(\alpha-1\right)\beta\right)\left(\left(2m-3n\right)F+q\right)/8\left(2m-n\right)^{2}$, it can be deduced that $\partial(\pi_{r}^{I^{*}}-\pi_{r}^{II^{*}})/\partial\rho<0$; $\partial(\pi_{t}^{I^{*}}-\pi_{t}^{II^{*}})/\partial\rho\text{=}-\left(2I/3qn+qm-3qn/2\right)\left(\left(m-n\right)F+q\right)\left(\left(m-2n\right)F+q\right)m^{2}c_{r}\left(1+\left(\alpha-1\right)\beta\right)/64\left(2m-n\right)^{4}$, since $m>2n$, $\partial(\pi_{t}^{I^{*}}-\pi_{t}^{II^{*}})/\partial\rho<0$; , , it is obviously that and .

***Appendix H***

$\pi_{c}^{I^{*}}-\pi_{c}^{II^{*}}=G(F)/64(-n+2m)(m-n)(m-2n)$， since $m>2n$，we get $64(-n+2m)(m-n)(m-2n)>0$. The relationship between the magnitudes of $\pi_{c}^{I^{*}}$ and $\pi_{c}^{II^{*}}$ depends on $G(F)$， where $G(F)=(m-2n)(m-n)\left(AF^{2}+\left(-16mq-8qn\right)F\right)-4(2m-n)(m+n)q^{2}$, $A=-8\left(m-5n/4\right)^{2}+41n^{2}/2$. The solutions for $A=0$ are $m_{2}$ and $m_{3}$, respectively, with the exclusion of $m_{3}$. The solutions for $G(F)$ about $F$ are $F_{5}=\left((m-n)(m-2n)(2m+n)-\sqrt{2mn^{2}(m-n)(m-2n)(4m+n)}\right)q/(m-n)(m-2n)A/4$ and $F_{6}=\left((m-n)(m-2n)(2m+n)+\sqrt{2mn^{2}(m-n)(m-2n)(4m+n)}\right)q/(m-n)(m-2n)A/4$, respectively. When $2n<m<m_{2}$, we have $A>0$, $F_{5}<F_{6}$, and the quadratic function of $G(F)$ about F opens upward, $G(0)\text{=}-4(2m-n)(m+n)q^{2}<0$, so $F_{5}<0<F_{6}$. If $F>F_{6}$, then $\pi_{c}^{I^{*}}>\pi_{c}^{II^{*}}$. If $0<F<F_{6}$, then $\pi_{c}^{I^{*}}<\pi_{c}^{II^{*}}$. When $m>m_{2}$, we have $A<0$, and the quadratic function of $G(F)$ about $F$ opens downward, moreover, since $G(0)<0$, $F_{6}<0$ and $F>0$, we get $\pi_{c}^{I^{*}}<\pi_{c}^{II^{*}}$.

***Appendix I***

$\pi_{m}^{I^{*}}-\pi_{m}^{II^{*}}=H(F)/8(-n+2m)(m-n)(m-2n)$, since $m>2n$, we get $8(-n+2m)(m-n)(m-2n)>0$. The relationship between the magnitudes of $\pi_{m}^{I^{*}}$ and $\pi_{m}^{II^{*}}$ depends on $H(F)$, where $\text{H}\left(F\right)=\left(m-n\right)\left(m-2n\right)\left(AF^{2}+\left(-16mq-8nq\right)F\right)/4-q^{2}(2m^{2}+nm-n^{2})$. The solutions for $H(F)$ about $F$ are $F_{5}\text{ }$ and $F_{6}$, respectively. Consistent with the proof of Corollary 7, if $2n<m<m_{2}$ and $F>F_{5}$, then $\pi_{m}^{I^{*}}>\pi_{m}^{II^{*}}$; If $2n<m<m_{2}$ and $0<F<F_{5}$, then $\pi_{m}^{I^{*}}<\pi_{m}^{II^{*}}$; If $m>m_{2}$, then $\pi_{m}^{I^{*}}<\pi_{m}^{II^{*}}$.
